# Supplementary material for: The coenzyme B12 precursor 5,6-dimethylbenzimidazole is a flavin antagonist in Salmonella
Source: Microb Cell. 2023 Aug 17;10(9):178–94. doi: 10.15698/mic2023.09.803 (PMC10468695; doi:10.15698/mic2023.09.803)
Supplement: Supplementary file 1 [file mic-10-178-s01.pdf]

microbial cell

Research Article

www.microbialcell.com

The coenzyme B_{12} precursor 5,6-dimethylbenzimidazole is a flavin antagonist in *Salmonella*

Lahiru Malalasekara[1] and Jorge C. Escalante-Semerena[1,*]

[1] Department of Microbiology, University of Georgia, Athens USA.

* Corresponding Author:

Jorge C. Escalante-Semerena, 212C Biological Sciences Building, 120 Cedar Street, Athens, GA 30602 USA; T: +1 (706) 542-2651, F: +1 (706) 542-2815; E-mail: jcescala@uga.edu

ABSTRACT ***Salmonella enterica*** **subsp.** ***enterica*** **sv. Typhimurium str. LT2 (hereafter *S.* Typhimurium) synthesizes adenosylcobalamin (AdoCbl, CoB_{12})** ***de novo*** **only under anoxic conditions, but it can assemble the lower ligand loop (a.k.a. the nucleotide loop) and can form the unique C-Co bond present in CoB_{12} in the presence or absence of molecular oxygen. During studies of nucleotide loop assembly in *S.* Typhimurium, we noticed that the growth of this bacterium could be arrested by the lower ligand nucleobase, namely 5,6-dimethylbenzimidazole (DMB). Here we report** ***in vitro*** **and** ***in vivo*** **evidence that shows that the structural similarity of DMB to the isoalloxazine moiety of flavin cofactors causes its deleterious effect on cell growth. We studied DMB inhibition of the housekeeping flavin dehydrogenase (Fre) and three flavoenzymes that initiate the catabolism of tricarballylate, succinate or D-alanine in *S.* Typhimurium. Notably, while growth with tricarballylate was inhibited by 5-methyl-benzimidazole (5-Me-Bza) and DMB, growth with succinate or glycerol was arrested by DMB but not by 5-Me-Bza. Neither unsubstituted benzimidazole nor adenine inhibited growth of *S.* Typhimurium at DMB inhibitory concentrations. Whole genome sequencing analysis of spontaneous mutant strains that grew in the presence of inhibitory concentrations of DMB identified mutations effecting the *cycA* (encodes D-Ala/D-Ser transporter) and *dctA* (encodes dicarboxylate transporter) genes and in the coding sequence of the tricarballylate transporter (TcuC), suggesting that increased uptake of substrates relieved DMB inhibition. We discuss two possible mechanisms of inhibition by DMB.**

doi: *10.15698/mic2023.09.803*
Received *originally: 18.05.2023;*
in revised form: 27.07.2023,
Accepted *07.08.2023,*
Published *17.08.2023.*

Keywords*: cobamide nucleobases, 5,6-dimethylbenzimidazole, CoB12 biosynthesis, benzimidazole antimicrobials, flavoenzyme inhibitors.*

Abbreviations:
5-FOA - 5-fluoroorotate,
5-Me-Bza - 5-methylbenzimidazole,
Cbas – cobamides,
DMB – 5,6-dimethylbenzimidazole,
EtBr – Ethidium bromide,
Fre – flavin reductase,
PMF – proton motive force,
Sdh – succinate dehydrogenase,
TSS – transcription start site.

INTRODUCTION

Benzimidazoles are aromatic compounds that are used as chemical scaffolds for the synthesis of antifungal, antiviral, antihelminth and anticancer agents [1, 2]. Because of the structural similarity of benzimidazoles to purines, these molecules can interfere with enzymes involved in DNA processing, hence, they are used as anticancer drugs [2-8]. Benzimidazoles can also be incorporated into nucleic acids suggesting that this family of compounds could be mutagenic [9]. Equally important is the use of benzimidazole derivatives that target different cellular processes in bacteria and fungi [10-13]. Therefore, a better understanding of the mode of action of a naturally occurring benzimidazole could provide insights into new strategies for the synthesis of more efficacious antimicrobials and anticancer agents.

Benzimidazole derivatives are used by bacteria and archaea to synthesis cobamides (Cbas), which are members of the family of cyclic tetrapyrroles known as 'The Pigments of Life' [14]. Cbas are central to the metabolism of cells from all domains of life, including humans, and unlike other members of this family of compounds, Cbas have axial ligands on both planes of the cyclic tetrapyrrole ring (**Fig. 1**). Although the nucleobases found in Cbas vary [15], the nucleobase relevant to the work reported herein is 5,6-dimethylbenzimidazole (hereafter DMB), which is the lower ligand in the Cba known as cobalamin [16]. Not all prokaryotes that synthesize Cbas incorporate DMB as the nucleobase, and if they do, its synthesis occurs via different pathways. For example, shown in **Figure 1A** is the conversion of $FMNH_2$ into DMB by the aerobic DMB synthase (BluB) enzyme (a.k.a. flavin destructase, EC 1.12.11.79) in a

series of complex chemical steps [17-21]. A BluB homologue is not present in *S.* Typhimurium, nor are the enzymes of the pathway found in the anaerobic bacterium *Eubacterium limosum* [22]. However, it is known that *S.* Typhimurium synthesizes DMB, but the pathway used by this bacterium is yet to be described [23]. **Figure 1B** shows the schematic of the late steps of adenosylcobalamin (a.k.a. AdoCbl, CoB_{12}) biosynthesis, which involves the incorporation of DMB into the final coenzyme structure. These late steps consist of four reactions, one reaction activates the nucleobase to generate an a-ribotide (catalyzed by CobT), a second reaction (catalyzed by CobU) activates adenosylcobinamide-phosphate (AdoCbi-P) into AdoCbi-GDP, a third reaction (catalyzed by CobS) condenses the a-ribotide and Ado-Cbi-GDP to yield adenosylcobalamin-5'-phosphate (AdoCbl-5'P), and the final reaction (catalyzed by CobC) dephosphorylates the substrate to yield the final product, CoB_{12}.

During studies of CobT substrate specificity [24, 25], we noticed that DMB could inhibit cell growth of *S.* Typhimurium. The observed inhibition was effective regardless of the carbon and energy source when the concentration of DMB in the medium was in the low mM range.

Here we investigated why DMB inhibits in *S.* Typhimurium. Briefly, we determined that the structural similarities of the substituted benzene ring of DMB with the isoalloxazine moiety of flavins is responsible for the deleterious effects of this CoB_{12} precursor. Results of *in vitro* and *in vivo* analyses led us to conclude that DMB interferes with the metabolism of *Salmonella* by outcompeting flavins that are substrates, but in the case of flavin prosthetic groups, we suggest that the inhibition caused by DMB may be due to *pi* stacking. In both scenarios the catalytic efficiency of the enzyme is reduced.

RESULTS

Growth inhibition caused by DMB is carbon source dependent and its effect is similar to the one exerted by a known flavoenzyme inhibitor

We observed that growth of wild-type *S.* Typhimurium in LB medium was inhibited as a function of the concentration of DMB, with growth being completely arrested when the concentration of DMB reached 3 mM (**Fig. 2A**). In contrast, *S.* Typhimurium growth was unaffected in medium supplemented with adenine (Ade, 3 mM) in lieu of DMB. We performed a parallel experiment using minimal medium supplemented with different carbon sources (**Fig. 2B**). Results obtained with minimal medium mimicked those obtained with LB medium, that is, the growth rate of *S.* Typhimurium decreased as a function of DMB concentration in the medium, with growth ceasing ~2-3 mM DMB when cells were grown in glucose, ribose, or glycerol as the carbon/energy source (**Fig. 2B**, left panel). Notably, DMB toxicity was more pronounced in minimal medium supplemented with either succinate, tricarballylate or D-alanine (**Fig. 2B**, left panel). When the latter carbon/energy sources were used, 1 mM DMB was sufficient to abrogate cell growth. This was a ~2-3-fold increase in DMB toxicity relative to LB + DMB medium. We noted that the catabolism of succinate, tricarballylate and D-alanine all start with dehydrogenations catalyzed by flavin-dependent enzymes,

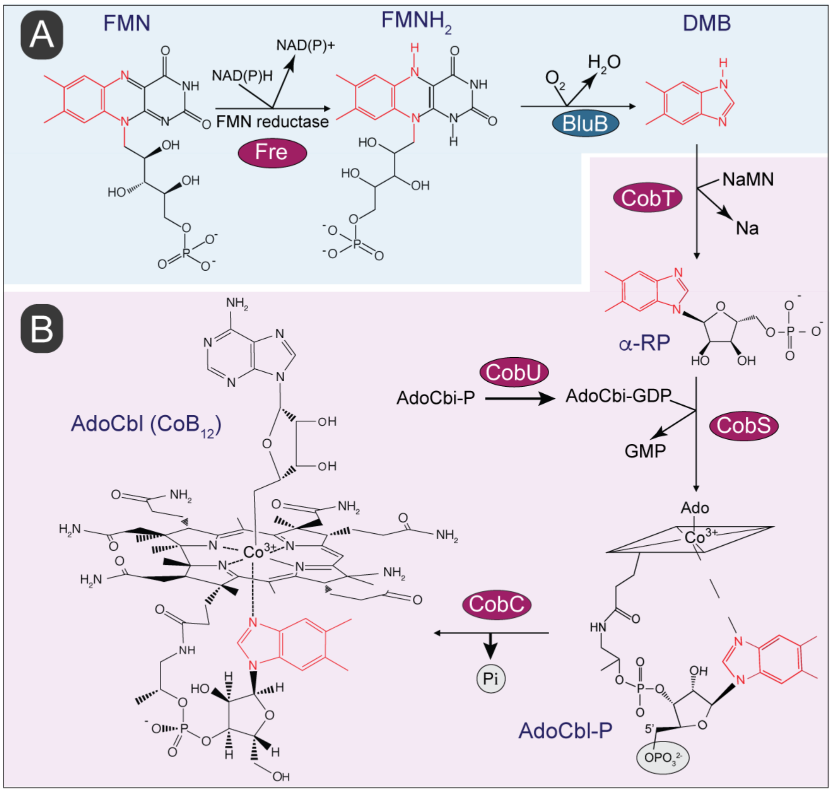

FIGURE 1: Biosynthesis of 5,6-dimethylbenzimidazole (DMB) and its incorporation into coenzyme B_{12} (CoB_{12}). (A) Aerobic pathway of DMB biosynthesis involves oxidative fragmentation of reduced flavin mononucleotide ($FMNH_2$) by DMB synthase, BluB. Atoms of the isoalloxazine ring and ribityl tail of $FMNH_2$ converted into DMB are shown in red. **(B)** In *S.* Typhimurium incorporation of DMB into CoB_{12} is initiated by its activation into the alpha ribotide, α-ribazole-5'-phosphate (α-RP) followed by condensation of activated intermediates and dephosphorylation to yield biologically active CoB_{12}. Abbreviations: FMN, flavin mononucleotide; NAD(P), nicotinamide adenine dinucleotide (phosphate); NaMN, nicotinate mononucleotide; Na, nicotinate; AdoCbi-GDP, adenosylcobinamide- guanosine diphosphate. GMP, guanosine monophosphate; AdoCbl-P, adenosylcobalamin-phosphate; AdoCbl, adenosylcobalamin; CobT, NaMN:DMB phosphoribosyltransferase; CobS, AdoCbl-5' phosphate synthase; CobC, adenosylcobalamin phosphatase; Fre, NAD(P)H:flavin reductase. Enzyme homologs presence in *S.* Typhimurium are shown in red circles. The rhomboid structure with the Co^{3+} ion in the middle is a cartoon of the complex cyclic tetrapyrrole ring shown at the end of the pathway.

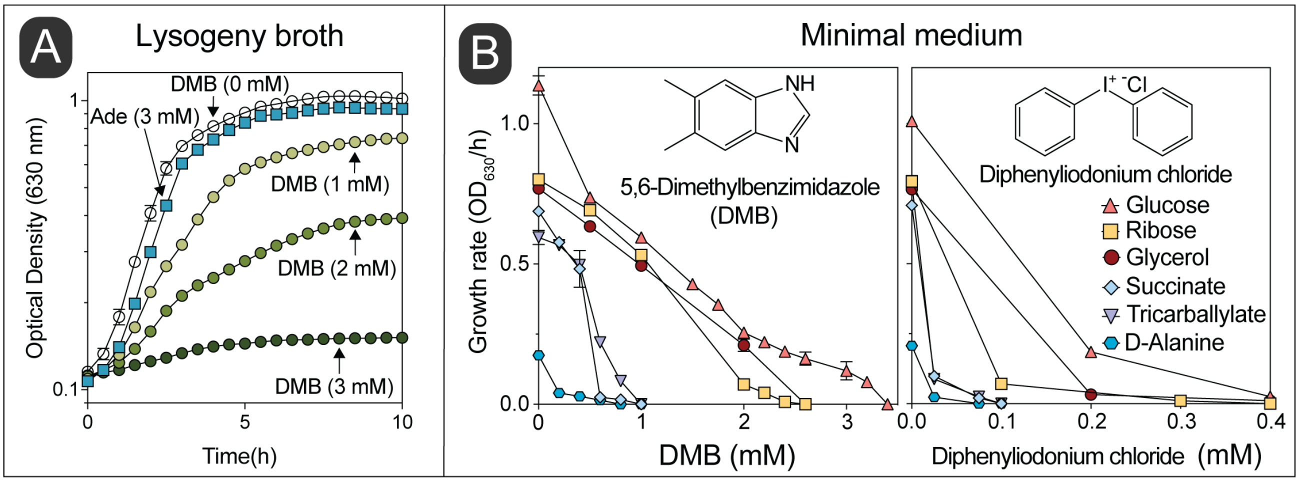

FIGURE 2: The inhibitory effect of DMB mimics that of a flavin antagonist. (A) Wild-type *S.* Typhimurium was grown in lysogeny broth with different concentrations of DMB or 3 mM of adenine. **(B)** Growth rates of *S.* Typhimurium grown in NCE minimal medium provided with different carbon sources and supplemented with different concentrations of DMB or diphenyliodonium chloride were calculated as described in *Materials and Methods*. Error bars represent standard deviation of three technical replicates and each experiment was performed thrice; a representative set of data are presented. Every data point has error bars, which if not shown it means the bars were smaller than the symbol.

hence, we hypothesized that DMB could be an antagonist of flavins. To explore this idea, we tested the effect of the known flavin inhibitor diphenyliodonium chloride, which yielded a similar pattern of carbon source-dependent growth inhibition (**Fig. 2B**, right panel) [26, 27]. Diphenyliodonium chloride was a more potent inhibitor than DMB under the conditions tested (~10-fold higher potency).

Consistent with the growth inhibition profile, which depended on the carbon and energy source provided, we also noticed that cell survival increased in minimal medium supplemented with glucose compared to medium supplemented with succinate, when challenged with an equimolar level of DMB (Fig. S1).

Analogs of DMB are not as toxic

To test the contributions of the methyl groups of DMB to its toxicity, we performed growth inhibition studies in medium supplemented with 5-methylbenzimidazole (5-Me-Bza), 5-methoxybenzimidazole (5-MeO-Bza), benzimidazole (Bza), 5-hydroxybenzimidazole (5-OH-Bza), and 4,5-dimethylphenylenediamine (DMPDA). As shown in **Figure 3**, DMB was the most potent growth inhibitor (**Fig. 3**, red diamonds). Remarkably, the absence of the methyl substituent at position 6 of the benzene ring resulted in cell growth when grown in glycerol or succinate as the main carbon source (**Fig. 3**, orange hexagons). However, 5-Me-Bza inhibited cell growth with tricarballylate reaching only an OD_{630} of ~0.3. For comparisons, the maximum OD_{630} in the absence of any supplement was ~ 0.8, and in medium supplemented with DMB the highest OD_{630} was ~0.2. When the medium contained either succinate or tricarballylate, the onset of exponential growth was comparable among cultures growing in the presence of different bases. However, when cells were grown in glycerol, we observed that the lag phase and the maximum cell density were negatively affected in medium supplemented with a nucleobase. In minimal glycerol medium growth was delayed for ~ 7 h when supplemented with 5-Me-Bza. 6-Me-Beza was not tested because it was not commercially available.

DMB is a competitive inhibitor of FMN

Considering the structural similarity of DMB to a part of the isoalloxazine moiety of flavins and given the importance of this structure to its toxicity, we hypothesized that DMB was a competitive inhibitor for flavins *in vitro*. To test this idea, we used NAD(P)H:flavin reductase (Fre) from *S.* Typhimurium, which catalyzes the reduction of FMN or FAD using electrons from reduced pyridine dinucleotides (NAD(P)H, **Fig. 1A**) [28]. In this experiment we used FMN as the flavin co-substrate for Fre. We observed inhibition of the enzyme activity with increasing concentrations of DMB (**Fig. 4A**, shades of gray bars *vs* white bar). In comparison, 5-Me-Bza and adenine **(Fig. 4A**, purple bar, green bar, respectively) were less inhibitory than DMB at equimolar concentrations. To investigate the mechanism of inhibition by DMB, we quantified the enzyme activity as a function of FMN at different concentrations of DMB. A double reciprocal plot showing the results of this experiment indicated a typical competitive inhibition kinetic with an apparent K_i of 1.9 mM ($\pm$ 3.5 µM; **Fig. 4B**).

DMB dissipates the proton motive force (PMF)

Because some flavoenzmes contribute to the proton motive force (PMF), we speculated that DMB could dissipate the PMF under growth conditions where flavoenzymes substantially contribute to the generation of the PMF. To test this idea, we used an ethidium bromide (EtBr) accumulation assay described elsewhere [29]. In this assay accumulation of EtBr inside the cell is used to indirectly measure the PMF. EtBr is an UV-fluorescent dye that stains DNA, and it is actively exported by the cell via a PMF-driven efflux pump [30]. Cells with a reduced PMF would accumu-

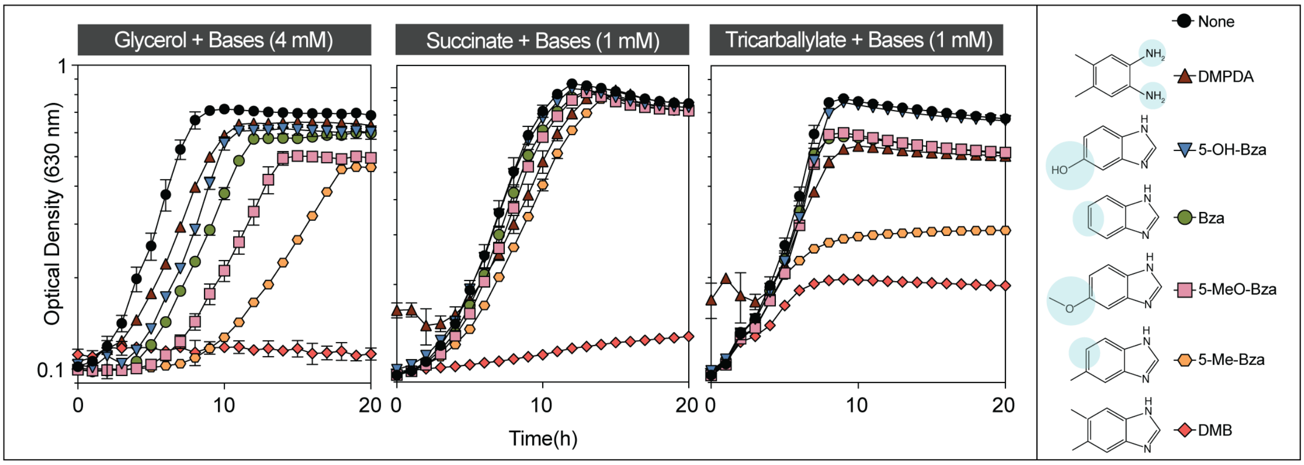

FIGURE 3: Toxicity of DMB and analogs. Wild-type *S.* Typhimurium was grown in NCE minimal medium supplemented with glycerol, succinate or tricarballylate as the main source of carbon and energy as described in *Materials and Methods*. Different bases were supplemented at 1 or 4 mM final concentration as indicated. Error bars represent standard deviation of three technical replicates and each experiment was completed with three biological replicates, with a representative graph shown. Error bars smaller than the symbols are not shown. Abbreviations: DMPDA, 4,5-dimethyl-1,2-phenylenediamine; 5-OH-Bza, 5-hydroxybenzimidazole; Bza, benzimidazole; 5-MeO-Bza, 5-methoxybenzimidazole; 5-Me-Bza, 5-methylbenzimidazole; DMB, 5,6-dimethylbenzimidazole. Structural differences of these bases in comparison to DMB are shaded in blue.

late EtBr as a function of time with the concomitant increase in fluorescence. When we grew *S.* Typhimurium in succinate as the main carbon and energy source and exposed mid-log phase cells to increasing concentrations of DMB, we measured a gradual increase in the rate of EtBr accumulation (**Fig. 5**, gray shaded bars *vs* white bar). When cells were exposed to $\geq$ 6 mM DMB for 15 min, intracellular EtBr accumulation was substantial ($p <$ 0.0047). In contrast, no increase in fluorescence was observed when cells were exposed to 8 mM 5-Me-Bza and a minimal effect was observed by the addition of 8 mM adenine (**Fig. 5**, purple, green bars, respectively). We used the *bona fide* PMF dissipator carbonyl cyanide-m-chlorophenylhydrazone (CCCP) as a positive control (**Fig. 5**, red bar). Supporting our hypothesis, we observed that the effect on PMF by DMB was higher in cells grown in succinate or tricarballylate compared to cells grown in glucose as the main carbon and energy source (Fig. S2).

Increased substrate uptake confers resistance to DMB

To determine the mode of DMB toxicity, we isolated mutants that grew when the culture medium was supplemented with inhibitory levels of DMB. We isolated spontaneous DMB resistant strains on minimal medium containing D-alanine, succinate or tricarballylate as carbon and energy source. The genome of DMB^R strains was sequenced to locate the causative mutations (**Table 1**). Remarkably, out of the ten strains sequenced, nine had mutations that affected the expression of the gene encoding the transporter for the carbon source used in the original selection. DMB resistance in each of these strains was specific to the original carbon source isolated, for example, a strain resistant to DMB on D-alanine minimal medium, was still sensitive to DMB on medium containing tricarballylate or succinate (**Table 1**).

The gain-of-function mutation in strain JE26530 was mapped to be 77 bp upstream of the transcription start site (TSS, labeled +1 in **Fig. 6A**) of the D-alanine transporter gene *cycA* (**Table 1**) [31]. Using RT-qPCR, we determined that this mutation caused a ~1.73-fold increase in *cycA* transcription compared to the wild-type *S.* Typhimurium strain (**Fig. 6B**). Hook *et al.*, showed binding of the global regulator Crp to this region of the chromosome in *E. coli,* and this region also contained a putative binding site for the nucleoid associated protein IHF (**Fig. 6A**) [32]. Notably, overexpression of *cycA* from the chromosome or a plasmid was sufficient for cells to become resistant to DMB (**Fig. 6C**, gray vs blue squares). At present, we do not know how the A-to-T mutation in strain JE26530 (**Table 1**) upregulates *cycA* expression; this question will be answered in future studies. To further verify that the alluded mutation in strain JE26530 resulted in increased uptake of D-alanine, we tested the sensitivity of strain JE26530 to D-cycloserine, an antibiotic known to be taken up by the CycA transporter [33]. **Figure 6D** shows results of an experiment performed with two strains overexpressing *cycA*. The chromosome of strain (JE26530) contains the A-to-T mutation in the *cycA* promoter (**Fig. 6A**). A second strain (JE26754) carries a plasmid in which *cycA* was cloned under the control of the arabinose inducible P_{araBAD} promoter [34]. Our results showed that the DMB^R strain (JE26530) was more sensitive to D-cycloserine than the parent strain (**Fig. 6D** red triangles vs green circles), and that ectopic expression of *cycA* (strain JE26754) also increased the sensitivity to D-cycloserine (**Fig. 6D**, gray *vs* blue squares).

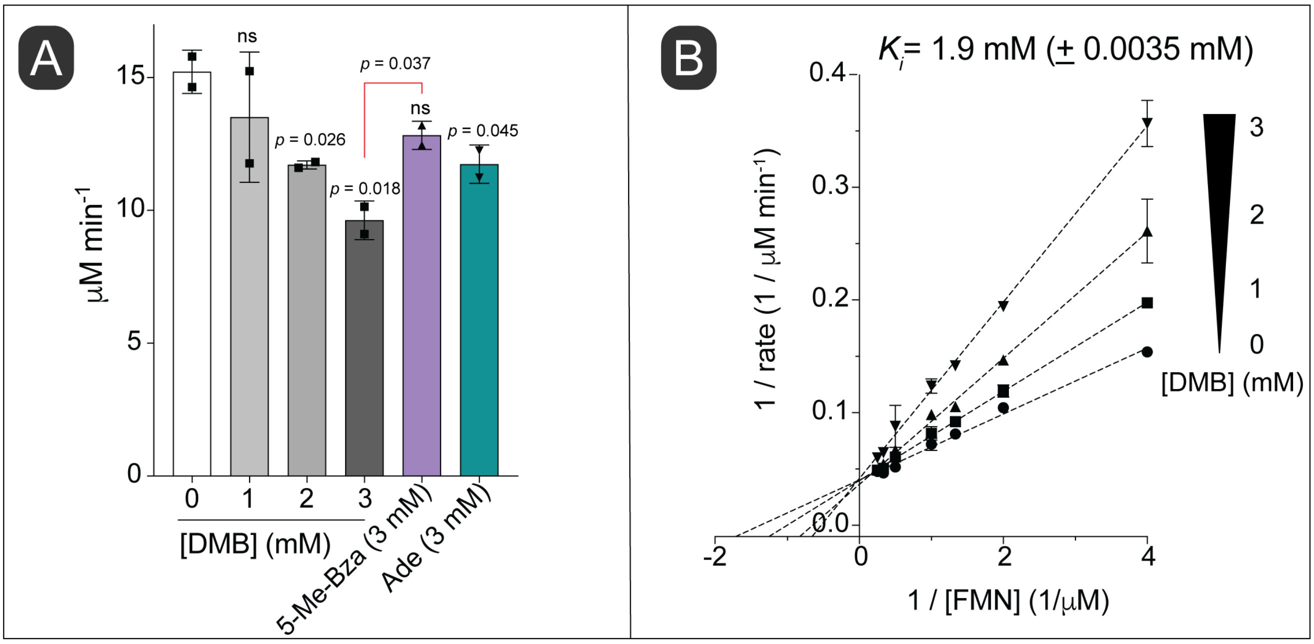

FIGURE 4: DMB is a competitive inhibitor of the NAD(P)H:flavin reductase (Fre) enzyme. (A) Initial reaction velocities were calculated using purified *S.* Typhimurium Fre enzyme as described in *Materials and Methods*. Reaction rates at a fixed concentration of FMN (1 mM) and different concentrations of DMB or other bases (as indicated) are shown. **(B)** Double-reciprocal plot of Fre initial rates at varying concentrations of FMN and different fixed concentrations of DMB as indicated. Error bars represent standard deviation of three technical replicates and each experiment was replicated twice, with a representative graph shown. K_i was calculated by a competitive inhibition model fit using Prism (GraphPad, v9). Standard deviation of two independent experiments is shown. An unpaired Student's *t* test was performed to determine statistical significance; ns, not significant. Statistical compassions were performed against the no-treatment control unless otherwise shown in the Figure.

Strains JE26529 and JE26837 were independently isolated from NCE minimal medium containing succinate as the main carbon and energy source plus DMB (1 mM). As shown in **Figure 7B** (red triangles), strain JE26529 grew well in the presence of inhibitory concentrations of DMB. Whole genome sequencing showed that both strains contained C-to-T changes at 26 bp downstream from the TSS (labeled +1 in **Fig. 7A**) of the dicarboxylate transporter gene *dctA*, whose function is required for the uptake of dicarboxylates, including succinate [35]. The above-mentioned mutation was mapped to the 5'-untraslated region (5'-UTR) of the *dctA* transcript. Like the results obtained with *cycA,* cells grown in succinate became resistant to DMB when *dctA*$^+$ was overexpressed *in trans* (**Fig. 7B**, blue vs gray squares). To further support the idea that an increase in succinate uptake would result in resistance to DMB, we took advantage of the fact that DctA is known to transport 5-fluoroorotate (5-FOA), a known inhibitor of pyrimidine biosynthesis [35]. We used 5-FOA to indirectly measure the efficiency of DctA function by comparing the sensitivity of DMB^S and DMB^R strains to FOA. Consistent with our hypothesis that the C-to-T mutation increased *dctA*$^+$ expression, we observed high sensitivity to 5-FOA in strain JE26529 (DMB^R) compared to the parent *S*. Typhimurium strain (DMB^S; **Fig. 7C**, red triangles vs green circles).

We also obtained DMB^R derivatives of *S.* Typhimurium when cells were exposed to inhibitory levels of DMB while growing with tricarballylate as the main carbon and energy source. Interestingly, all mutations identified by whole genome sequencing of DMB^R strains isolated from tricarballylate minimal medium were located within the coding sequence of the tricarballylate transporter gene, *tcuC* (**Table1**, **Fig. 8A**). The alluded mutations changed a glycine residue into serine or aspartate residues (i.e., G173S, G173D), or a leucine residue into serine (L135S). The structure of TcuC has not been solved, however, we used the AlphaFold algorithm [36, 37] to get an idea of what the possible location of the above-mentioned residues may be in a three-dimensional model of the tricarballylate transporter. This structure suggested alluded mutations are likely located within the transmembrane channel of TcuC (Fig. S3). To test the effect of these residues on DMB resistance, we performed site directed mutagenesis and constructed a plasmid carrying a *tcuC* allele encoding TcuC^{G173S}. When *tcuC* was expressed from this plasmid, cells gained resistance for DMB (**Fig. 8B**, purple diamonds). However, we also learned that plasmid-encoded expression of the wild-type allele of *tcuC* was sufficient for the resistance (**Fig. 8B**, blue squares).

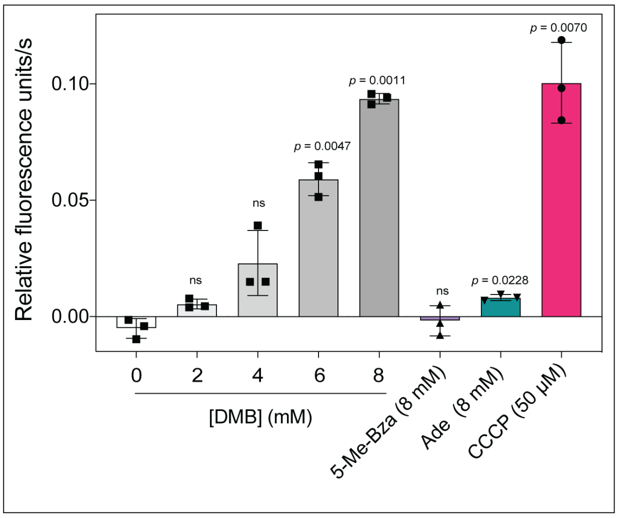

FIGURE 5: DMB impairs proton motive force in *S.* Typhimurium. Cells were grown in NCE minimal medium supplemented with succinate (30 mM) and acetate (0.5 mM) to an OD_{600} of ~0.5 and added nucleobases or CCCP as indicated; cultures were incubated for 15 min at 37°C with shaking. Cells were stained with EtBr, cultures were irradiated with 530-nm light, and fluorescence emitted at 600 nm was monitored over 4 min. The rate of EtBr accumulation is expressed as relative fluorescence units/s. Results are provided as the mean from three biological replicates, with each experiment containing technical triplicates. Error bars denote the standard deviation of three biological replicates. A paired Student's *t* test was performed compared to the no treatment control to determine statistical significance; ns, not significant. Abbreviations: DMB, 5,6-dimethylbenzimidazole; 5-Me-Bza, 5-methylbenzimidazole; Ade, adenine; CCCP, carbonyl cyanide *m*-chlorophenolhydrazone.

Not all transporters can overcome the sensitivity to DMB

To test whether resistance to toxic levels of DMB was due to the increase in carbon source uptake regardless of the type of the carbon source provided, we tested whether *S.* Typhimurium could become resistant to DMB when either the gene encoding the glycerol uptake facilitator protein, *glpF*, or the *rbsACB* genes encoding the ribose ABC transporter were ectopically overexpressed in cells growing in minimal medium supplemented with glycerol or ribose. As shown in Figure S4A, neither a higher level of GlpF nor RbsABC resulted in DMB resistance. We confirmed that ectopically expressed *rbsACB* resulted in functional RbsACB transporter by introducing this plasmid and demanding growth of a *Salmonella rbsACB* mutant on ribose as the sole source of carbon (Fig. S4B).

***In vitro* evidence that DMB inhibits succinate dehydrogenase**

The above results suggested that DMB might interfere with FAD-dependent dehydrogenases that catalyze the first step in the catabolism of succinate, D-alanine and tricarballylate. The alluded dehydrogenases included succinate dehydrogenase (Sdh complex), D-alanine dehydrogenase (DadA) and tricarballylate dehydrogenase (TcuA). Given the above results, we surmised that increased intracellular substrate concentration could alleviate the inhibition by DMB. To test this hypothesis, we tested the activity of the Sdh complex as a function of succinate concentration during exposure to an inhibitory concentration of DMB. However, our double reciprocal plot did not indicate a competition between succinate and DMB for the active site. Instead, we observed a noncompetitive inhibition (**Fig. 9A**). This revealed that DMB could bind to both substrate-free and substrate-bound versions of the Sdh complex. To verify this idea, we tested the inhibition of Sdh activity by adding DMB before vs after the reaction was initiated. We observed inhibition of the reaction by DMB in both cases, in

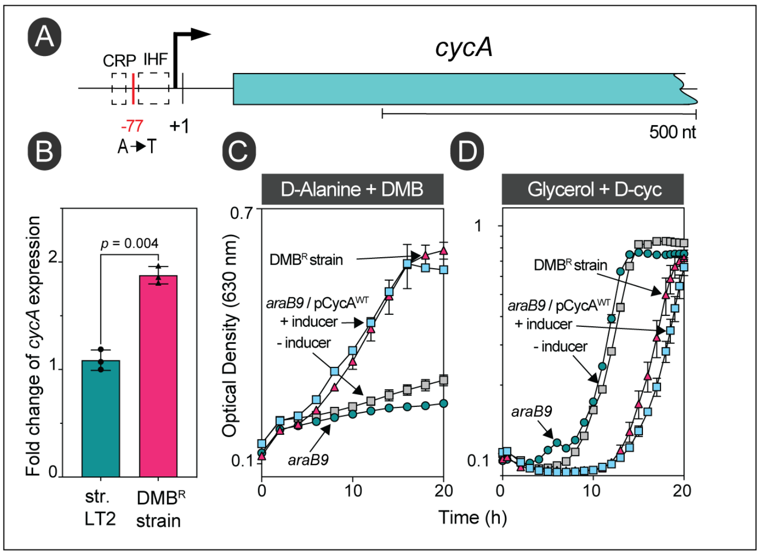

FIGURE 6: Overexpression of the wildtype allele of *cycA* provides resistance to DMB during growth with D-alanine. (A) Location of the gain-of-function mutation in strain JE26530. This strain was originally isolated from D-alanine minimal medium supplemented with DMB (0.7 mM). The *cycA* transcription start site is denoted by + 1 [31]. **(B)** RT-qPCR of *cycA* expression in wildtype *S.* Typhimurium (JE9426) or its DMB resistant derivative (JE26530) grown with D-alanine plus DMB (0.2 mM). Fold change values were calculated by normalizing relative expression values to the lowest *cycA*+ expression. Error bars represent standard deviation of three biological replicates. **(C)** Growth analysis to study DMB resistance of cells growing with D-alanine. Cells were grown in NCE minimal medium supplemented with D-alanine (20 mM), glucose (0.5 mM) and DMB (0.7 mM). **(D)** The DMB resistant strain has a higher sensitivity for D-Cycloserine (D-Cyc). Cells were grown in NCE minimal medium supplemented with glycerol (20 mM), with or without D-Cyc (20 µM) as indicated. L-(+)-Arabinose (0.5 mM) was used to induce of plasmid-borne *cycA*+ in all experiments. Strain JE26530 was the DMB resistant strain used in these studies; str. LT2 is an abbreviation of the wildtype strain of *S.* Typhimurium. Error bars represent standard deviation of three technical replicates and each experiment was completed with three biological replicates, with a representative graph shown.

TABLE 1. Mutations that revert DMB inhibition. Characterization of DMB resistant gain-of-function (GOF) strains. All mutant strains were derivatives of *S.* Typhimurium strain JE9426, and details of their isolation are described in *Materials and Methods*.

Strain ID	Carbon source used	Location of the mutation	Cellular function of affected gene	Nucleotide change	Location/annotation	OD_{600} after 24 h (SD)		
						D-alanine + DMB	Succinate + DMB	Tricarbal-lylate+ DMB
JE26530	D-alanine	Promoter of *cycA*	D-serine/D-alanine/glycine transporter	A→T	-77 nt from TSS	0.48 (±0.02)	NG	NG
JE26843	D-alanine	RBS of *cycA*	D-serine/D-alanine/glycine transporter	A→G	+71 nt from TSS	0.50 (±0.04)	NG	NG
JE26529, JE26837	Succinate	5' UTR of *dctA*	C4-dicarboxylate transporter	C→T	+26 nt from TSS	NG	0.65 (±0.03)	NG
JE26835	Succinate	CDS of *yohM*	Nickel/cobalt efflux pump	$(CGACCA)_{13\rightarrow12}$	coding (D441-446/867 nt)	NG	0.76 (±0.06)	NG
JE26838	Succinate	5' UTR of *dctA*	C4-dicarboxylate transporter	G→A	+31 nt from TSS	NG	0.70 (±0.03)	NG
JE26839, JE26841	Tricarbal-lylate	CDS of *tcuC*	Tricarballylate transporter	G→A	G173S (GGT→AGT)	NG	NG	0.49 (±0.03)
JE26840	Tricarbal-lylate	CDS of *tcuC*	Tricarballylate transporter	T→C	L135S (TTA→TCA)	NG	NG	0.45 (±0.01)
JE26842	Tricarbal-lylate	CDS of *tcuC*	Tricarballylate transporter	G→A	G173D (GGT→GAT)	NG	NG	0.51 (±0.01)

Abbreviations: SD, standard deviation; NG, no growth (OD_{600} < 0.25); TSS, transcription start site; RBS, ribosome binding site; UTR, untranslated region; CDS, coding sequence.

fact the inhibition of an initiated reaction was higher (**Fig. 9B**). The results suggested DMB did not compete with succinate for binding to the Sdh complex.

Cells respond to exogenous flavins under DMB stress

Given the data supporting that DMB is a flavin antagonist, we reasoned that increasing the level of FMN/FAD in the cell would reverse the phenotype. To test this idea, we added riboflavin (the immediate precursor of FMN) to the medium and noticed a slight improvement in cell growth (**Fig. 10A**, filled black circles *vs* squares) even though *S.* Typhimurium does not have a known riboflavin transporter. To circumvent the absence of a riboflavin transporter, we ectopically expressed the *bona fide* riboflavin transporter PnuX from *Corynebacterium glutamicum* [38], and repeated the experiment. We observed the same relative improvement in yield but neither the growth rate nor the final cell density approached that of cultures that were never exposed to DMB (**Fig. 10A**, open symbols). Since this was a negative result, we confirmed that PnuX was functional by testing the susceptibility of the *pnuX*-expressing *S.* Typhimurium strain to roseoflavin, a toxic analog of riboflavin [39]. As seen in **Figure 10B** PnuX was functional since medium supplemented with roseoflavin affected the growth rate of *S.* Typhimurium when *pnuX* was heterologously expressed. However, we noticed that when cells were grown on succinate as the main carbon and energy source, they could no longer respond to exogenous flavins upon exposure to DMB (Fig. S5B).

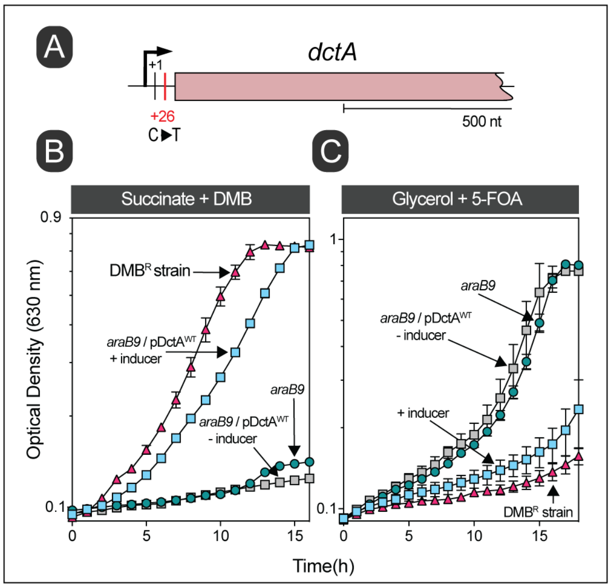

FIGURE 7. Overexpression of *dctA* is sufficient for DMB resistance in succinate. (A) Chromosomal location of the gain-of-function mutation in strain JE26529. This strain was isolated from succinate minimal medium supplemented with DMB (1 mM). The transcription start site is denoted as + 1 [35]. **(B)** Growth analysis to study DMB resistance in succinate is shown. Cells were grown in NCE minimal medium supplemented with succinate (30 mM), acetate (0.5 mM) and DMB (1 mM). **(C)** DMB resistant mutation increased the sensitivity of cells for 5-fluoroorotate (5-FOA). Cells were grown in NCE minimal medium supplemented with glycerol (20 mM) with or without providing 5-FOA (1 µg/mL) as indicated. L-(+)-Arabinose was used at a 0.5 mM final concentration for induction. DMB resistant mutant represented here is JE26529. Error bars represent standard deviation of three technical replicates and each experiment was completed with three biological replicates, with a representative graph shown.

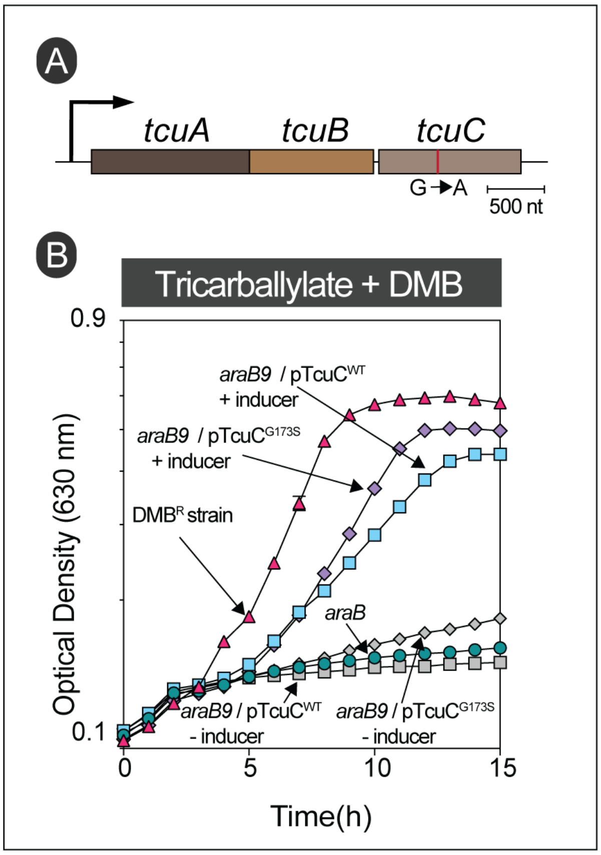

FIGURE 8: Overexpression of *tcuC* is sufficient for DMB resistance in tricarballylate. (A) relative genetic context depicting gain of function mutation in strain JE26839. This strain was originally isolated from tricarballylate minimal medium contained DMB (1 mM). Tricarballylate utilizing genes are arranged into an operon in *Salmonella*, these genes include *tcuA* encoding tricarballylate dehydrogenase, *tcuB* encoding FADH2; quinone oxidoreductase and *tcuC* encodes for tricarballylate transporter [51]. **(B)** Growth analysis to study DMB resistance in tricarballylate is shown. Cells were grown in NCE minimal medium supplemented with tricarballylate (20 mM), glucose (0.5 mM) and DMB (1 mM). L-(+)-Arabinose was used at a 0.5 mM final concentration for induction. DMB resistant mutant represented here is JE26839. Error bars represent standard deviation of three technical replicates and each experiment was completed with three biological replicates, with a representative graph shown.

DISCUSSION

In this paper we investigated the molecular basis for the toxicity of the nucleobase of CoB_{12}, i.e., 5,6-dimethylbenzimidazole (DMB) in *S.* Typhimurium.

DMB targets flavoenzymes

The data presented in **Figure 2** show that DMB inhibits *S.* Typhimurium cell growth under either rich or minimal medium and that DMB inhibition mimics the effect of a *bona fide* flavoenzyme inhibitor. Notably, DMB was a much better inhibitor in minimal medium when the catabolism of the carbon source provided was initiated by a flavoenzyme, (e.g., succinate, D-alanine or tricarballylate). Under those conditions the exogenous DMB concentration needed to fully arrest *S.* Typhimurium growth was < 1 mM. It is interesting that succinate dehydrogenase (SdhABCD), tricarballylate dehydrogenase (TcuA) and D-Ala dehydrogenase (DadA) are either in close association with the membrane (i.e., TcuA, DadA) [40, 41] or are integral membrane proteins (SdhABCD) [42], making their interactions with a hydrophobic molecule like DMB more likely to occur during transit across the cell membrane, thus blocking the generation of reducing power, reducing the PMF (**Fig. 5**) and ultimately ATP generation by the PMF-driven ATP synthase.

Rosenberg *et al*. showed that increased levels of intracellular succinate is a signal for *Salmonella* to activate its virulence, and thus important to survive within macrophages [43]. In this study we demonstrated disturbance of succinate metabolism in the presence of µM levels of DMB *in-vivo* (**Fig. 2**). This suggests that biosynthesis of DMB, and its interference on succinate metabolism might be an added advantage for pathogenesis of *Salmonella*.

The methyl substituents of the benzimidazole (Bza) ring are critical to DMB toxicity

Our data support the idea that the methyl groups at positions 5 and 6 of DMB determine how inhibitory the DMB analog is to *S.* Typhimurium. Additionally, we noticed a pronounced effect of 5-Me-Bza when cells were grown on tricarballylate (**Fig. 3**). We speculate that these differences in the cytotoxicity of Bza derivatives is likely due to varying affinity of the molecule for the target site of the flavoenzyme. We believe the observed effects, particularly between 5-Me-Bza and DMB, are unlikely due to different rates of diffusion across the cell membrane, even though DMB is slightly more hydrophobic than 5-Me-Bza. A carbon source dependency, and the specific structure of a benzimidazole derivative suggest that flavoenzymes are the target of DMB. Carbon source and benzimidazole structure specificity also suggest that the main cause for the growth inhibition is likely due to the interference of DMB with flavoenzymes, not due to nonspecific effect(s) on cell physiology. However, we do not rule out the possibility that DMB could interfere with other cellular processes at low mM levels. We tested whether the hydrophobicity of DMB could interfere with the integrity of the cell envelope, by challenging cells with vancomycin, an antibiotic that does not readily permeate through an intact Gram-negative outer membrane [44]. We did not observe any increased susceptibility to vancomycin in the presence of sub-lethal levels of DMB (Fig. S6).

Increased influx of substrate effectively reverses DMB inhibition

The results of all the suppressor analyses we performed were consistent with an increased influx of substrate, i.e., D-alanine, succinate or tricarballylate. In the case of D-alanine, results of qPCR analysis showed that the level of expression of the *cycA* gene encoding the D-Ala:H^+ symporter increased by almost 2-fold in a strain that was no longer sensitive to inhibitory levels of DMB in the medium (**Fig. 6B**). We surmise that the increase in *cycA* expression allowed more substrate to enter the cell, kept the enzyme

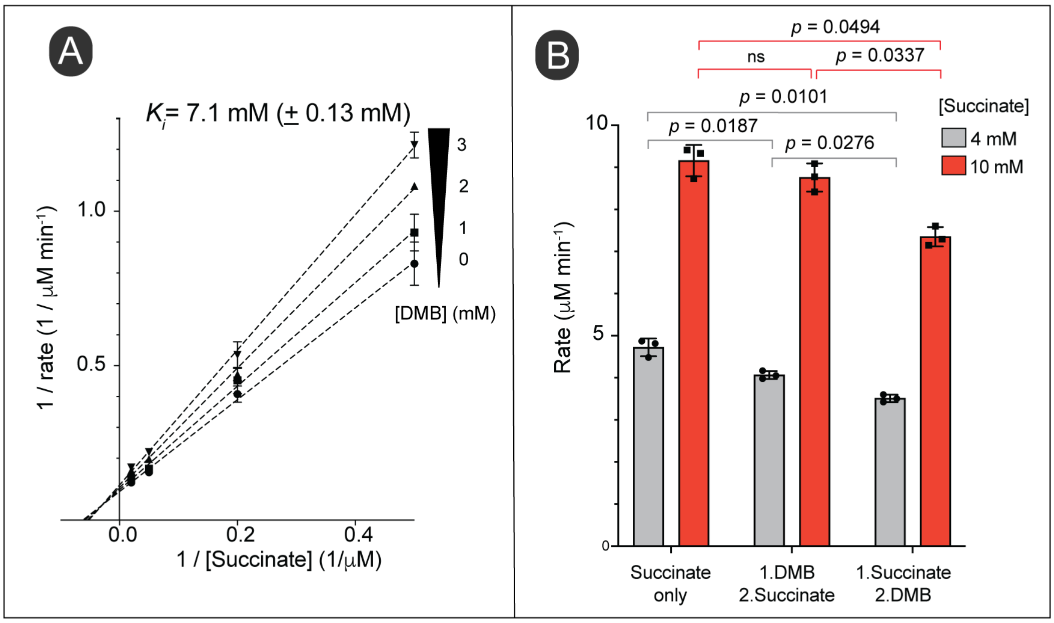

FIGURE 9: DMB inhibits succinate dehydrogenase *in vitro*. (A) Double-reciprocal plot of initial reaction velocities of succinate dehydrogenase at varying concentrations of succinate and different fixed concentrations of DMB as indicated. Error bars represent standard deviation of two technical replicates and each experiment was replicated twice, with a representative graph shown. The K_i was calculated by a noncompetitive inhibition model fit using Prism (GraphPad, version 9). Standard deviation of two independent experiments is shown. **(B)** Effect of the order of adding DMB into the reaction mixture for its inhibition. Reactions were set up adding all the reaction components as described in *Materials and Methods* except succinate and DMB; incubated at RT for 5 min. DMB (3 mM) or succinate was added to the reaction as indicated and further incubated for 30 s at room temperature. The second missing component (either succinate or DMB) was added and progress of the reaction was monitored as described in *Materials and Methods*. We verified the all the reactions were within the linear range of the reaction progress when initial velocities were calculated (data not shown). A paired Student's *t* test was performed to determine statistical significances; ns, not significant. Initial reaction rates were calculated using crude extracts of *S.* Typhimurium grown in NCE minimal medium supplemented with succinate as the main source of carbon and energy.

saturated to prevent DMB from entering the active site resulting in growth of the revertant strain in medium containing DMB (**Fig. 6C**). The A-to-T mutation in the promoter region of *cycA* is likely to be the causative lesion since it was the only single-nucleotide change detected after the entire genome of the revertant strain was sequenced. This conclusion is supported by the increased sensitivity of the DMB^R (JE26530) strain to D-cycloserine, an antibiotic that is translocated into the cell by CycA. It follows that an increase in CycA would result in increased D-cycloserine sensitivity regardless of whether the strain was sensitive or resistant to DMB, and that is what we observed for the parent strain (JE9426, DMB^S) and the DMB^R (JE26530) revertant (**Fig. 6D**).

Surprisingly, almost all the genomes of DMB^R strains we sequenced had mutations in the promoters/regulatory regions of genes encoding the transporters for D-alanine (*cycA*) and succinate (*dctA*), or in the coding sequence of the *tcuC* gene encoding the tricarballylate transporter TcuC. We suggest that higher DctA levels or a more efficient TcuC variant was synthesized in the respective DMB^R strain (**Table 1** and **Fig. 7, 8**). Notably, overexpression of *dctA* and *tcuC in trans* was sufficient to phenocopy DMB resistance (**Fig. 7, 8**), and as expected in the case of *dctA* overexpression, the DMB^R (JE26838) strain became more sensitive to 5-FOA, a cell inhibitor known to be transported by *dctA* (**Fig. 7C**).

Not surprisingly, DMB resistance was specific to the carbon source used for the isolation of revertant strains (**Table 1**), such a resistance to DMB was not due to a generalized effect of increased uptake of a carbon source since cells grown in glycerol or ribose were not resistant to DMB when the transporters for these carbon sources (*glpF, rbsACB,* respectively) were ectopically overexpressed (Fig. S4A).

Multiple mechanisms of inhibition by DMB

DMB is a competitive inhibitor of the cytosolic FMN reductase (Fre; **Fig. 4B**), but our data suggest that DMB can also be a non-competitive inhibitor (**Fig. 9A**). In *E. coli*, a close relative of *S.* Typhimurium, the FAD cofactor of Sdh is attached to residue H45 of the SdhA subunit of the Sdh complex [45-47]. We speculate that in cases like the Sdh complex, where the flavin cofactor is covalently attached to the enzyme, DMB may interfere with the substrate binding and/or efficient transfer of protons from substrate to FAD.

We observed that Sdh activity was inhibited by DMB with a high K_i (7.1 mM; **Fig. 9A and B**) in an apparently noncompetitive inhibition way. The pattern of Sdh inhibition suggested that DMB can bind to free and substrate-bound Sdh complexes [48], hence, we speculate that the structural similarities between DMB and the isoalloxazine moiety of FAD may allow *pi*-staking interactions in the active site of the enzyme that blocks catalytic turnover. This idea is not unprecedented, since a previous study by others has predicted formation of *pi*-staking interactions between a benzimidazole and a histidine residue of the active site of a sirtuin deacetylase [8]. A noncompetitive inhibition does not always rule out binding of the inhibitor to the active site. For instance, if an enzyme undergoes multiple transi-

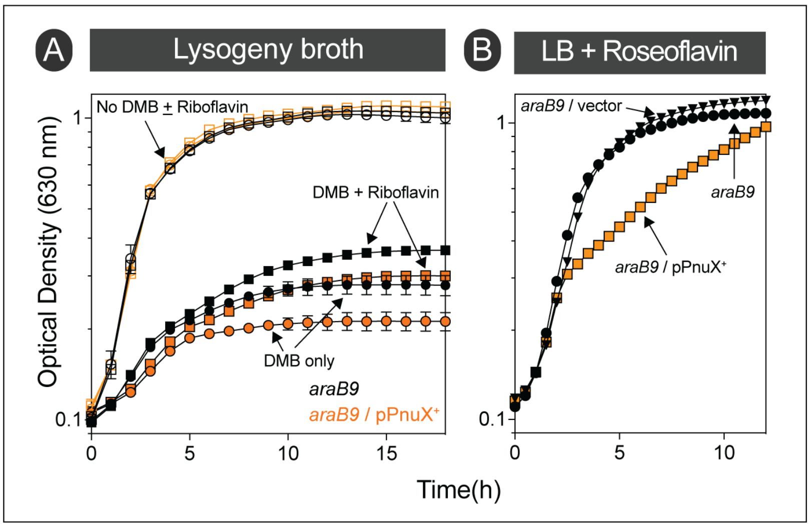

FIGURE 10: *S.* Typhimurium responds to exogenous riboflavin under DMB stress, but growth cannot be fully recovered. (A) Cells were grown in lysogeny broth with (squares) or without (circles) riboflavin (0.75 mM). Cultures with added DMB (2 mM) are shown in filled shapes. Black colored graphs represent *araB9* strain (JE10079) and orange graphs represent *ara9* /pPnuX+ (JE26910) strain harboring *C. glutamicum* riboflavin transporter gene *pnuX in trans*. **(B)** Heterologous expression of *C. glutamicum* riboflavin transporter gene *pnuX* increased its sensitivity to roseoflavin, a toxic analog of riboflavin; cultures were grown in lysogeny broth containing roseoflavin (500 µM). L-(+)-Arabinose (0.5 mM) was added to the medium to induce gene expression. Error bars represent standard deviation of three technical replicates and each experiment was completed with three biological replicates, with a representative graph shown**.**

tions during a single catalytic cycle and if some transition conformation(s) of the active site favor binding of the inhibitor, this would yield a non-competitive pattern of inhibition [49, 50]. This notion of DMB interference with the catalytic turnover of the Sdh complex, but not to replace the flavin from its active site was supported as we observed a lack of response to exogenous flavins in cells grown in succinate as the source of carbon under DMB stress (Fig. S5B). In addition, it is important to notice that all three transporters we discussed in this study (DctA, CycA and TcuC) are substrate:proton symporters, which implies their activity is dependent on PMF [51-53]. In this case, it is likely that a reduced PMF caused by DMB would decrease the uptake of the substrate, which in turn would lower the generation of reducing power creating a negative feedback loop. In addition, as we have shown with the activity of the Sdh complex, the inhibitory effect of DMB could magnified under sub-saturating levels of succinate (**Fig. 9B**, gray vs red bars in both cases of inhibition). As observed with the gain-of-function mutants described in this study *S*. Typhimurium can overcome the inhibitory effect of DMB by increasing the uptake of succinate.

Concluding remarks

We suggest that the structural similarities between DMB and flavins cause a redox problem for the cell when the concentration of exogenous DMB is sufficiently high for it to cross the cell membrane and reach inhibitory levels inside the cell. The data indicate that DMB could inhibit an enzyme which is in a transient association with flavins (*e.g.*, Fre) and flavoenzymes whose cofactor does not diffuse out of the active site (*e.g.*, SdhABCD). The antagonistic effect of DMB on flavoenzymes may be the major mode of toxicity on cellular growth. However, we cannot rule out other possible inhibitory effects on cell metabolism.

MATERIALS AND METHODS

Bacterial strains and chemicals.

All strains constructed were derivatives of *Salmonella enterica* subsp. *enterica* serovar Typhimurium strain LT2 (referred to as *S.* Typhimurium). All strains used in this study, including *E. coli* strains used for protein overexpression, are listed in **Table 2**. Unless otherwise indicated, chemicals were obtained from Sigma-Aldrich, and were used without further purification. 5-Hydroxybenzimidazole was purchased from Combi-Blocks Inc (San Diego, CA). Stock solutions of all the bases were prepared in dimethylsulfoxide (DMSO).

Culture media and growth studies

All strains were grown in lysogenic broth (LB, Difco) or no-carbon essential (NCE) minimal medium [54]. NCE medium was supplemented with $MgSO_4$ (1 mM), Wolfe's trace minerals (1×) [55] and glucose (11 mM), ribose (22 mM), glycerol (20 mM), succinate (30 mM), D-alanine (20 mM) or tricarballylate (20 mM) were used as carbon and energy sources at the stated concentrations. Medium supplemented with succinate also contained a low concentration of sodium acetate (0.5 mM), whereas glucose (0.5 mM) was added to the medium when D-alanine or tricarballylate was used as the carbon source. When used, antibiotics were added at the following concentrations: ampicillin, 100 µg/mL; chloramphenicol, 20 µg/mL; and kanamycin, 50 µg/mL. Cultures used as inoculant were grown overnight (~20 h) at 37°C in LB, and small samples (2%, v/v) were used to inoculate 196 µL of fresh medium placed in each well of a 96-well microtiter plate. L-(+)-Arabinose was used as an inducer wherever indicated. Microtiter plates (Falcon) were incubated at 37°C inside the temperature-controlled chamber of a microtiter plate reader (BioTek Instruments), and plates were continuously shaken using the medium setting of the instrument. Cell density was monitored at 630 nm, and data were analyzed using Prism software package v9 (GraphPad). Growth rate calculations were performed using online tools. The calculations include confidence intervals for Gompertz models (https://scott-h saunders.shinyapps.io/gompertz_fitting_0v2/). When taking the viable counts to determine the cell survival, appropriate dilutions were prepared using saline (0.9 % w/v NaCl), samples were plated on LB agar plates and plates incubated overnight (~20 h) at 37°C.

TABLE 2. Bacterial strains and plasmids used in this study.

Strain	Relative genotype	Source[a]
E. coli strains		
DH5a	Φ80d*lacZ*ΔM15 *recA1 endA1 gyrA96 thi-1 hsdR17* (r_{k}-, m_{k}^{+}) *supE44 relA1 deoR* Δ(*lacZYA-argF*) *U169 phoA*	Laboratory collection
C41(λDE3)	*ompT hsdS* ($r_{B}m_{B}$) *gal* λ(DE3)	Laboratory collection
S.* Typhimurium[b] *strains		
JE9426	*Salmonella enterica* subsp. *enterica* serovar Typhimurium LT2 reference genome	Gift from from D. M. Downs
JE26530	*cycA1*	
JE26843	*cycA2*	
JE26529	*dctA81*	
JE26835	*yohM200*	
JE26837	*dctA81*	
JE26838	*dctA82*	
JE26839	*tcuC58*	
JE26840	*tcuC59*	
JE26841	*tcuC58*	
JE26842	*tcuC60*	
JE10079	*araB9*	
JE26754	*araB9* / pCYCA1	
JE26906	*araB9* / pDCTA1	
JE26964	*araB9* / pGLPF1	
JE26998	*araB9* / pTCU97	
JE26999	*araB9* / pTCU98	
JE26910	*araB9* / pCgPnuX2	
JE27053	*araB9* / pRBS1	
JE26616	*araB9* / pCV1	
JE23873	*araB9* / pCV3	Laboratory collection
JE10813	*araB9* / pKD46	Laboratory collection
JE27069	*araB9 rbs81::kan*$^{+}$	
JE27082	*araB9 rbs81::kan*$^{+}$ / pRBS1	
JE27083	*araB9 rbs81*::*kan*$^{+}$ / pCV1	
**Plasmid**	**Description**	**Source[a]**
pCV1	Complementation vector P_{araBAD} *bla*$^{+}$	[58]
pCV3	Complementation vector P_{araBAD} *cat*$^{+}$	[58]
pCYCA1	*S.* Typhimurium *cycA*$^{+}$ cloned into pCV3	
pDCTA1	*S.* Typhimurium *dctA*$^{+}$ cloned into pCV1	
pTCU97	*S.* Typhimurium *tcuC*$^{+}$ cloned into pCV1	
pTCU98	*S.* Typhimurium *tcuC58*$^{+}$ encoding $TcuC^{G173S}$ in pCV1	
pGLPF1	*S.* Typhimurium *glpF*$^{+}$ cloned into pCV1	
pRBS1	*S.* Typhimurium *rbsACB*$^{+}$ cloned into pCV1	
pCgPnucX2	*Cornyebacterium glutamicum pnuX*$^{+}$ cloned into pCV3	Laboratory collection
pTEV18	TEV protease-cleavable, *N*-terminal H_6 tag overexpression vector	[58]
pFRE4	*S.* Typhimuriuma *fre*$^{+}$ cloned into pTEV18	
pKD4	Template plasmid carrying *kan*+ gene	[59]
pKD46	Expresses l Red recombinase system	[59]

[a] Unless otherwise stated strains and plasmids were constructed during this work.

[b] *S.* Typhimurium is an abbreviation of *Salmonella enterica* subspecies *enterica* serovar Typhimurium strain LT2

Selection for DMB resistant mutants and whole genome sequencing

Spontaneous DMB resistant strains were isolated by spreading ~2 x 10^8 cells per plate of wild-type *S.* Typhimurium (strain JE9426) grown in LB on NCE minimal medium supplemented with agar (1.5 % w/v) and DMB (0.7-1 mM). Plates were incubated at room temperature (~25°C) for 4-5 days. Samples were taken from isolated colonies and streaked three consecutive times on LB plates. DMB resistance of isolated mutants was verified by growing them in liquid NCE minimal medium supplemented with DMB (0.7-1 mM).

For whole genome sequencing, we isolated genomic DNA (gDNA) from strains JE26529 (*dctA81*) and JE26530 (*cycA1*) grown overnight in 4 mL of LB, gDNA was precipitated using isopropanol as describe elsewhere [56]. DNA quality was analyzed using a Qubit 4 fluorometer with the DNA broad range

(BR) assay kit (Invitrogen). High-throughput genome sequencing was carried out on the Illumina platform NovaSeq PE150 at Novogene Corporation Inc (Sacramento, CA). Alignments were generated by mapping the reads to the reference genome of *S.* Typhimurium (NCBI accession no NC_003197.2 & NC_003277.2) using Integrative Genomic Viewer (IGV_2.11.9).

Extraction of gDNA, whole genome sequencing and mutations calling for rest of the mutant strains were performed at SeqCenter sequencing facility (Pittsburgh, USA). For the whole genome sequencing of these strains Illumina 200 Mbp package was used, and variant calling was performed against the reference genome of *S.* Typhimurium (NCBI accession no NC_003197.2 & NC_003277.2).

Test for antibiotic susceptibility

An overnight culture (~20 h) of *S.* Typhimurium (JE9426) grown in LB was diluted to a ~1 x 10^8 CFU/ mL in saline (0.9% w/v NaCl) and cells (150 µL) were spread onto Muller Hinton agar plates (Sigma-Aldrich) supplemented with DMB or ethylenediaminetetraacetic acid (EDTA, 250 µM). Plates were dried at room temperature for 10 min, sterilized E-strips of vancomycin (Liofilchem) were placed, and plates were incubated at 37°C for ~18-20 h. Minimum inhibitory concentration (MIC) was determined as the value where the zone of inhibition intersected with the strip.

Plasmid construction

All plasmids used in this work are listed in **Table 2**. Primers used in this study were synthesized by Integrated DNA Technologies, Inc. (IDT,Coralville, IA]), and are listed **Table 3**. Genes *cycA* (*stm4398*), *dctA* (*stm3614*), and *tcuC* (*stm0689*) *glpF* (*stm4087*) and *rbsACB* (*stm3882-4*) were amplified using Phusion high fidelity polymerase (Thermo Fisher Scientific). PCR products were analyzed on 0.8% agarose (w/v, Apex Bio-Reserach) gels after staining with ethidium bromide (Sigma, 0.5 µg/mL) and cleaned up using the Wizard SV gel and PCR cleanup system (Promega). The *cycA* amplicon was cloned into pCV3 vector whereas *dctA*, *tcuC*, *glpF, and rbsACB* amplicons were cloned into pCV1 followed by BspQI restriction digestion as described elsewhere [57, 58]. Following the same procedure *fre* (STM3979) was cloned into pTEV18 [58] for overexpression. After transformation of cloned plasmids into *E. coli* DH5a cells, followed by selecting with appropriate antibiotics a colony PCR screen was performed to identify plasmids carrying an insert with the expected size. Plasmids were isolated using the Wizard Plus SV miniprep kit (Promega) and sequence of the cloned region was verified at the sequencing facility of Eton Bioscience, Inc. Plasmids pCYCA1, pDCTA1, pTCU97, pGLPF1 and pRBS1 were electroporated into an *S.* Typhimurium *araB9* strain (JE10079) for growth studies.

Site directed mutagenesis was performed to change position 173 from a glycine to serine in TcuC, using plasmid pTCU97 as template. Primers for mutagenesis was designed using PrimerX (https://www.bioinformatics.org/primerx/, Table 3) and PCR amplification followed by DpnI restriction digestion to cleave the template was performed following the QuickChange site directed protocol (Stratagene), but changed the extension temperature to 68°C and extension time of 2.5 min per kb in our amplification reactions. Mutations were confirmed by Sanger sequencing (Eton Bio, NC). The resulting

TABLE 3. List of primers used in this study.

Primer	Sequence
Primers used for plasmid construction	
270.Fre.pTEV18_F	NNGCTCTTCNTTCATGACAACCTTAAGCTGTAAAG
271.Fre.pTEV18_R	NNGCTCTTCNTTATTAAATAAATGCGAACGCATCG
287.cycA.BspQI_F	NNGCTCTTCNTTCATGGTAGATCAGGTAAAAGTCGC
288.cycA.BspQI_R	NNGCTCTTCNTTATTAACGCATTCCCGCCATC
319.dctA_BspQI_F	NNGCTCTTCNTTCATGAAAACCTCTCTGTTCAAAAG
320.dctA_BspQI_R	NNGCTCTTCNTTATTAGGAGGAAATTTCGTGCG
343.glpF.BspQI_F	NNGCTCTTCNTTCATGAGTCAAACATCAACCTTAAAAG
344.glpF.BspQI_F	NNGCTCTTCNTTATTACAGCGAAGCATTTTGTTG
351.tcuC.BspQI_F	NNGCTCTTCNTTCATGGCACAACACACACC
352.tcuC.BspQI_R	NNGCTCTTCNTTATCAGGCTTTATTTTCTGCCG
373.rbsA.BspQI_F	NNGCTCTTCNTTATTACTGCTTGACCAGTTC
374.rbsB.BspQI_R	NNGCTCTTCNTTATTACTGCTTGATGACCAGTTTC
**Primers used for site-directed mutagenesis**	
339.TcuC.G173S_F	GTTGTTGCCGCGTTGATTAGTTATAGCCTGAATATC
340.TcuC.G173S_R	GATATTCAGGCTATAACTAATCAACGCGGCAACAAC
**Primers used for chromosomal gene deletion**	
378.W_rbsA-C_F	CTGAACCTGATCCCGCAGTTGACCATTGCGGAAAACGTGTAGGCTGGAGCTGCTTC
379.W_rbsA-C_R	AACATCCGGATGCGCGGTTAGCAGGTTCTGCATGACCATATGAATATCCTCCTTAG
**Primers used for RT-qPCR**	
251.cycA.qPCR_F	CCATCTATAAGATGCCGCTCGG
252.cycA.qPCR_R	CGTATCGTCTTCCAGCGTCAG
257.gyrA.qPCR_F	GGGCGTTATCCTTATCCGCAC
258.gyrA.qPCR_F	GCCGTCGATAGCGTCGAGTTC

plasmid (pTCU98) was electroporated into an *S.* Typhimurium *araB9* strain (JE10079) for growth studies.

Strain construction

In-frame chromosomal deletion of ribose ABC type transporter genes (*rbsACB*) was constructed using phage λ Red recombinase system described elsewere [59, 60]. Briefly, an amplicon containing kanamycin resistance cassette with flanking homologous regions to *S*. Typhimurium *rbsA* and *rbsB* was amplified and purified following the same procedure described under *Plasmid construction* section. For this purpose, pKD4 plasmid [59] was used as the template and primers contained homologous regions are listed in Table 3. Amplicon (~1 µg) was electroporated into electrocompetent *araB9* strain harboring plasmid pKD46, which encoded λ Red recombinase (JE10813). Cells were then recovered with 1 mL of Super Optimal Broth (SOB) supplemented with glucose (20 mM) for 1 h at 37°C and plated on LB agar (1.5 %) supplemented with kanamycin (50 µg/mL). Recombination on Kan^R resistant colonies was verified by a colony PCR and chromosomal deletion was reconstructed into strain JE10079 (*araB9*) using P22-mediated transduction of the drug marker of a selected colony.

Overproduction and purification of Fre

Overproduction and purification of *S*. Typhimurium Fre enzyme was performed with modifications to a protocol described elsewhere [28]. Briefly, the plasmid encoding H_6-Fre (pFRE4) was transformed into *E*. *coli* strain C41 (λDE3). This overexpression strain was grown in a 1L of Terrific Broth (Cold Spring Harbor Laboratory Protocols) with ampicillin at 37°C with shaking (180 rpm) to an OD_{600} of ~ 0.9, induced with isopropyl-b-D-thiogalactopyranoside (IPTG) to a final concentration of 0.4 mM and further incubated for 2 h under the same conditions. Cells were harvested by centrifugation at 6,000 x *g* for 15 min at 4°C and the pellet was stored at -80°C until used.

The cell pellet was resuspended in 25 mL of binding buffer [20 mM Tris-HCl at pH 7.9 containing NaCl (500 mM) and imidazole (5 mM)] supplemented with protease inhibitor phenylmethylsulfonylfluoride (PMSF, 1 mM), lysozyme (1 mg/mL), and DNase (0.1 mg/mL). Cells were lysed on ice for four, 30-s cycles of sonication (2 s on, 2 s off) at 50% amplitude on a Qsonica sonicator and lysate was centrifuged at 40,000 x *g* for 2 h at 4°C using an Avanti J-25I refrigerated floor centrifuge equipped with a JA-25.25 rotor (Beckman Coulter). The clarified cell free extract was loaded onto 3-mL column volume (CV) of nickel-nitrilotriacetic acid (Ni-NTA) affinity resin (HisPur, Thermo Fisher Scientific). The column was washed with 10 CVs of bind buffer followed by 6 CVs of wash buffer [20 mM Tris-HCl at pH 7.9 containing NaCl (500 mM) and imidazole (60 mM)]. Protein was eluted off the column with two CVs of elution buffer [20 mM Tris-HCl at pH 7.9, containing NaCl. (500 mM) and imidazole (400 mM)]. Fractions containing eluted protein were pooled, and protein concentration was quantified spectrophotometrically (NanoDrop 1000; Thermo Fisher Scientific) using the molar extinction coefficient (34380 $M^{-1}cm^{-1}$) and molecular mass (29.58 kDa). To cleave H_6- tag, recombinant tobacco etch virus (rTEV) protease [61] was added at a ratio of 1 mg TEV: 75 mg protein and dialyzed overnight and then two times of 3-h each against the binding buffer at 4 °C. Dialyzed protein was re-loaded onto the Ni-NTA column and repeated the purification with 4 CVs of binding buffer, 3 CVs of washing buffer and 2CVs of elution buffer. Fraction containing tag-less Fre were pooled and dialyzed against Tris-Cl buffer [50 mM at pH 8 containing ethylenediaminetetraacetic acid (EDTA, 1 mM) and *tris*(2-carboxyethyl)phosphine hydrochloride (TCEP, 0.5 mM)] with decreasing amounts of NaCl down to 250 mM at 4°C. A final dialysis was performed against the latter buffer containing 10% glycerol and 150 mM of NaCl at 4°C for 3 h. Protein was quantified after dialysis using Bradford protein assay (Bio-Rad Laboratories) according to the manufacturer's instructions. Protein was flash frozen in liquid nitrogen and stored at -80°C until use.

Determination of inhibition kinetics of flavin reductase, Fre

To determine the inhibition kinetics of flavin reductase, activity of the enzyme was measured upon exposure to different concentrations of DMB. Activity was determined under aerobic conditions by measuring the oxidation of NADH at 340 nm as described elsewhere [62]. Reactions were set up in a 96-well plate and performed at the room temperature. Reaction mixture contained Fre enzyme (30 nM) and NADH (180 µM) in N-(2-hydroxyethyl)piperazine-N9-(3-propanesulfonic acid) (HEPPS) buffer (200 mM, pH 7.5). Different fixed concentrations of DMB were added to the reactions and incubated for 5 min. Reactions were started by the addition of varying concentrations of FMN, and absorbance at 340 nM was measured for 10 min at 30 s intervals using a Spectramax Plus UV-visible spectrophotometer (Molecular Devices). Reaction rate was determined by measuring by simple linear regression and using the molar absorptivity for NADH (6,220 M^{-1} cm^{-1}). The inhibition constant (K_i) was calculated by a competitive inhibition model fit using Prism (GraphPad, v9) software and the statistical significance was determined using unpaired *t* test.

Preparation of crude extracts for Sdh assay

Crude extracts were prepared by growing wild-type *S.* Typhimurium (strain JE9426) in minimal medium supplemented with succinate as the main source of carbon and energy. Briefly, NCE minimal medium (200 mL) supplemented with succinate (30 mM) and sodium acetate (0.5 mM) was prepared as described under the *Culture media and growth studies* section. Overnight culture (~16-20 h) grown in LB was used to inoculate (2.5 % v/v) and cultures were harvested at early stationary phase (OD_{600} ~ 1) by centrifugation at 6,000 x *g* for 15 min at 4°C. Pellet was washed with saline (0.9% w/v NaCl) and stored at -80°C until used.

The cell pellet was resuspended in 20 ml of potassium phosphate buffer (50 mM, pH 7.5) supplemented with lysozyme (1 mg/mL), DNase (0.1 mg/mL) and a cocktail of protease inhibitors (1x, Pierce™ Protease Inhibitor Tablet). Cells were lysed by passing them three times through a cell disruptor (Constant Systems) set at 1.72 x 10^5 kPa. Cell lysate was centrifuged at 25,000 x *g* for 12 min at 4°C using an Avanti J-25I refrigerated floor centrifuge equipped with a JA-25.25 rotor (Beckman Coulter) to pellet down cell debris. Crude extract was concentrated using a Amicon Ultra-15 Centrifugal Filter Unit (10 kDa cutoff) and dialysis three times against potassium phosphate buffer (50 mM, pH 7.5) containing NaCl (100 mM) at 4°C. Final dialysis buffer was supplemented with glycerol (10% v/v). Total protein was quantified using Bradford protein assay (Bio-Rad Laboratories) and was flash frozen in liquid nitrogen and stored at -80°C until use.

Determination of inhibition kinetics of succinate dehydrogenase

Succinate dehydrogenase activity of the crude extract was measured by following the reduction of 2,6-dichlorophenol-indophenol (DCPIP) as described elsewhere [63]. In this assay oxidation of succinate was coupled to the reduction of DCPIP. Assay was set up in 96-well plate at room temperature. Reaction mixtures contained potassium cyanide (10 mM), phenazine methosulphate (0.5 mM), DCPIP (0.3 mM), in potassium phosphate buffer (50 mM, pH 7.5). Crude extract was added to a final total protein concentration of 0.4 mg/mL. Different fixed concentrations of DMB were added to the reactions and incubated for 5 min. When adding different concentrations of DMB to the reaction, different stock solutions were prepared to make the introducing volume of DMSO consistent among different reactions. Reactions were started by the addition of varying concentrations of succinate and coupled reduction of DCPIP was monitored at 600 nm for 15 min with 30 s intervals using a Spectramax Plus UV-visible spectrophotometer (Molecular Devices). When tested for order of addition of DMB to the reaction, a reaction mixture prepared either adding DMB or succinate, incubated 30 s at RT and added the missing reaction component (succinate or DMB) before start monitoring OD_{600}. Reaction rate was determined by linear regression and using the molar absorptivity for DCPIP (21,500 M^{-1} cm^{-1}) [64]. The inhibition constant (K_i) was calculated by noncompetitive inhibition model using Prism (GraphPad, v9) and the statistical significance was determined using paired *t* test.

RNA isolation

Strains JE9426 (wild-type) and JE26530 (DMB^R gain of function) were grown overnight in triplicate in LB and sub-cultured (5% v/v) into 5 mL of NCE minimal medium supplemented with $MgSO_4$ (1 mM), Wolfe's trace minerals (1x), D-alanine (20 mM), glucose (0.5 mM) and DMB (0.2 mM). Cultures were grown with shaking (180 rpm) at 37°C to an optical density at 600 nm of ~0.5, and cells were harvested by centrifugation at 3,220 x *g* for 10 min using an Eppendorf 5810 R benchtop centrifuge. Pellets were flash-frozen in liquid nitrogen and stored at -80°C until further processing. RNA was isolated following the RNAsnap protocol [65]. Pellets were resuspended in 150 µL of boil solution (18 mM EDTA, 0.025% [w/v] SDS, 95% [v/v] RNA-grade formamide, 1% [v/v] 2-mercaptoethanol) and were vigorously vortexed. Pellets were incubated at 95°C for 7 min and centrifuged at 16,000 x *g* for 5 min at room temperature. The supernatant (150 µL) was transferred to a fresh tube and diluted by adding of 400 µL of RNase-free water. RNA precipitation was initiated by adding of 50 µL of sodium acetate (3 M, pH 5.2; final concentration, 0.3 M) and 1650 µL of ice-cold absolute ethanol (100%). Samples were vortexed and incubated overnight at -80 °C. After incubation, samples were centrifuged at 16,000 x *g* for 30 min at 4°C, and supernatant was decanted off. Pellet was wash by adding 300 µL of ice-cold ethanol (70% [v/v]), centrifuged at 8,000 x *g* for 5 min at 4°C to remove ethanol and pellets were allowed to air dry. RNA pellets were resuspended 100 µL in RNase-free water. Insoluble contaminants were removed by centrifugation at 16,000 x *g* for 1 min and supernatant (90 µL) was transferred to a new tube. A DNAse treatment was performed using Turbo DNA-free kit according to the manufacturer's instructions (Thermo, Fisher Scientific). Following the DNase treatment, samples were centrifuged at 10,000 x *g* for 1.5 min, and 90 µL was transferred into a new tube. A final sodium acetate-ethanol precipitation was performed as described above. The yield and quality of purified RNA was analyzed using a Qubit 4 fluorometer with the RNA broad range (BR) assay kit and the RNA IQ assay kit, respectively (Invitrogen). Samples with an RNA IQ value of 7 or higher were used for RT-qPCR experiments.

cDNA synthesis and quantitative reverse transcription PCR (RT-qPCR)

cDNA templates for RT-qPCR were synthesized using the iScript cDNA synthesis kit (Bio-Rad Laboratories) according to the manufacturer's protocol with a final RNA concentration of 12.5 ng/µL in the reaction mixture. Quantitative PCR reactions were set up in a 96-well plate using FastSYBR green master mix (Applied Biosystems) following manufacturer's instructions. Each reaction mixture (20 µL) contained 10 µL FastSYBR green master mix, gene-specific primers (500 ng each; **Table 3**), and cDNA (25 ng). Real-time data acquisition was performed using a 7500 Fast real-time PCR system (Applied Biosystems). Cycle threshold (C_T) values were normalized to the *gyrA* gene [66]. Three biological replicates of each strain were analyzed with three technical replicates in each. These normalized values (ΔC_T) were transformed using $2(e\text{-}\Delta C_T)/10^6$ [67] and were reported as the gene expression ratio of the mutant strain (JE26530) to the WT strain (JE9426). Statistical significance was determined using unpaired *t* test in Prism (GraphPad, v9) software.

EtBr accumulation assay

Effect of DMB on the PMF was measured by an EtBr accumulation assay as outlined elsewhere [29]. Strain JE9426 (wild-type) was grown overnight (~20 h) in LB and sub-cultured into (5% v/v) NCE minimal medium as described under culture media and growth studies section). Cultures were grown with shaking (180 rpm) at 37°C to an optical density at 600 nm of ~0.5 and treated for 15 min with various concentrations of DMB or other bases under the same conditions. Carbonyl cyanide *m*-chlorophenylhydrazone (CCCP; 50 µM final) was used as positive control for the assay. Cultures (200 µL) were transferred into a black, round-bottom 96-well microtiter plate, and EtBr was added to a final concentration of 6.25 µM. Relative fluorescence was monitored immediately after addition of EtBr using a BioTek Gemini fluorescent plate reader for 4 min at 530 nm and 600 nm excitation and emission wavelengths, respectively. The rate of relative fluorescence was determined by simple linear regression and significance was determined by paired *t* test using Prism (GraphPad, version 9) software.

ACKNOWLEDGMENTS

This work was supported by NIH MIRA grant R35-130399 to J.C.E.-S. We thank Ronnie L. Fulton for discussions of this work.

SUPPLEMENTAL MATERIAL

All supplemental data for this article are available online at www.microbialcell.com.

CONFLICT OF INTEREST

The authors declare no conflict of interest with this work.

COPYRIGHT

© 2023 Malalasekara and Escalante-Semerena. This is an open-access article released under the terms of the Creative Commons Attribution (CC BY) license, which allows the unrestricted use, distribution, and reproduction in any medium, provided the original author and source are acknowledged.

Please cite this article as: Lahiru Malalasekara and Jorge C. Escalante-Semerena (**2023**). The coenzyme B12 precursor 5,6-dimethylbenzimidazole is a flavin antagonist in Salmonella. **Microbial Cell** 10(9): 178-194. doi: 10.15698/mic2023.09.803

REFERENCES

1. Spasov AA, Yozhitsa IN, Bugaeva LI, Anisimova VA (**1999**). Benzimidazole derivatives: Spectrum of pharmacological activity and toxicological properties (a review). **Pharm Chem J** 33(5): 232-243. doi: 10.1007/BF02510042

2. Law CSW, Yeong KY (**2021**). Benzimidazoles in drug discovery: A patent review. **Chem Med Chem** 16(12): 1861-1877. doi: 10.1002/cmdc.202100004

3. Chen AY, Yu C, Bodley A, Peng LF, Liu LF (**1993**). A new mammalian DNA topoisomerase I poison Hoechst 33342: Cytotoxicity and drug resistance in human cell cultures. **Cancer Res** 53(6): 1332-1337. PMID: 8383008

4. Soderlind KJ, Gorodetsky B, Singh AK, Bachur NR, Miller GG, Lown JW (**1999**). Bis-benzimidazole anticancer agents: targeting human tumour helicases. **Anticancer Drug Des** 14(1): 19-36. PMID: 10363025

5. Ibrahim HA, Refaat HM (**2020**). Versatile mechanisms of 2-substituted benzimidazoles in targeted cancer therapy. **Future J of Pharm Sci** 6(1): 1-20. doi: 10.1186/s43094-020-00048-8

6. Kumar R, Singh G (**2022**). Substituted benzimidazoles as antibacterial and antifungal agents: A review. **Pharmacophore** 13(2): 41-55. doi: 10.51847/ySnvqCudRM

7. Vaithiyalingam M, Mohan Kumar R, Kamaraj C, Sugumar V, Manivannan N, Kadaikunnan S, Ghodake G (**2023**). Facile synthesis of benzimidazoles via oxidative cyclization of acyclic monoterpene aldehyde with diamines: Studies on antimicrobial and in vivo evaluation of zebrafish. **Chem Biodivers** 20(6): e202300315. doi: 10.1002/cbdv.202300315

8. Yoon YK, Ali MA, Wei AC, Shirazi AN, Parang K, Choon TS (**2014**). Benzimidazoles as new scaffold of sirtuin inhibitors: green synthesis, in vitro studies, molecular docking analysis and evaluation of their anti-cancer properties. **Eur J Med Chem** 83: 448-454. doi: 10.1016/j.ejmech.2014.06.060

9. Seiler JP (**1973**). The mutagenicity of benzimidazole and benzimidazole derivatives. II. Incorporation of benzimidazole into the nucleic acids of *Escherichia coli*. **Mutat Res** 17(1): 21-25. doi: 10.1016/0027-5107(73)90249-2

10. Wang YN, Bheemanaboina RRY, Cai GX, Zhou CH (**2018**). Novel purine benzimidazoles as antimicrobial agents by regulating ROS generation and targeting clinically resistant *Staphylococcus aureus* DNA groove. **Bioorg Med Chem Lett** 28(9): 1621-1628. doi: 10.1016/j.bmcl.2018.03.046

11. Sheng J, Nguyen PT, Baldeck JD, Olsson J, Marquis RE (**2006**). Antimicrobial actions of benzimidazoles against the oral anaerobes *Fusobacterium nucleatum* and *Prevotella intermedia*. **Arch Oral Biol** 51(11): 1015-1023. doi: 10.1016/j.archoralbio.2006.05.002

12. Ranjith PK, Rajeesh P, Haridas KR, Susanta NK, Row TN, Rishikesan R, Kumari NS (**2013**). Design and synthesis of positional isomers of 5 and 6-bromo-1-[(phenyl)sulfonyl]-2-[(4-nitrophenoxy)methyl]-1H-benzimidazoles as possible antimicrobial and antitubercular agents. **Bioorg Med Chem Lett** 23(18): 5228-5234. doi: 10.1016/j.bmcl.2013.06.072

13. Rao TN, AlOmar SY, Ahmed F, Albalawi F, Ahmad N, Rao NK, Rao MVB, Cheedarala RK, Reddy GR, Naidu TM (**2021**). Reusable nano-zirconia-catalyzed synthesis of benzimidazoles and their antibacterial and antifungal activities. **Molecules** 26(14): 4219. doi: 10.3390/molecules26144219

14. Battersby AR (**2000**). Tetrapyrroles: the pigments of life. **Nat Prod Rep** 17(6): 507-526. doi: 10.1039/b002635m

15. Chan CH, Escalante-Semerena JC (**2011**). ArsAB, a novel enzyme from *Sporomusa ovata* activates phenolic bases for adenosylcobamide biosynthesis. **Mol Microbiol** 81(4): 952-967. doi: 10.1111/j.1365-2958.2011.07741.x

16. Hodgkin DG, Pickworth J, Robertson JH, Trueblood KN, Prosen RJ, White JG (**1955**). The crystal structure of the hexacarboxylic acid derived from B12 and the molecular structure of the vitamin. **Nature** 176(4477): 325-328. doi: 10.1038/176325a0

17. Maggio-Hall LA, Dorrestein PC, Escalante-Semerena JC, Begley TP (**2003**). Formation of the dimethylbenzimidazole ligand of coenzyme B(12) under physiological conditions by a facile oxidative cascade. **Org Lett** 5(13): 2211-2213. doi: 10.1021/ol034530m

18. Campbell GR, Taga ME, Mistry K, Lloret J, Anderson PJ, Roth JR, Walker GC (**2006**). *Sinorhizobium meliloti bluB* is necessary for production of 5,6-dimethylbenzimidazole, the lower ligand of B12. **Proc Natl Acad Sci U S A** 103(12): 4634-4639. doi: 10.1073/pnas.0509384103

19. Gray MJ, Escalante-Semerena JC (**2007**). Single-enzyme conversion of $FMNH_2$ to 5,6-dimethylbenzimidazole, the lower ligand of B_{12}. **Proc Natl Acad Sci U S A** 104(8): 2921-2926. doi: 1073/pnas.0609270104

20. Taga ME, Larsen NA, Howard-Jones AR, Walsh CT, Walker GC (**2007**). BluB cannibalizes flavin to form the lower ligand of vitamin B12. **Nature** 446(7134): 449-453. doi. 10.1038/nature05611

21. Wang XL, Quan JM (**2011**). Intermediate-assisted multifunctional catalysis in the conversion of flavin to 5,6-dimethylbenzimidazole by BluB: A density functional theory study. **J Am Chem Soc** 133(11): 4079-4091. doi: 10.1021/ja1106207

22. Hazra AB, Han AW, Mehta AP, Mok KC, Osadchiy V, Begley TP, Taga ME (**2015**). Anaerobic biosynthesis of the lower ligand of vitamin B12. **Proc Natl Acad Sci U S A** 112(34): 10792-10797. doi: 10.1073/pnas.1509132112

23. Mattes TA, Escalante-Semerena JC (**2017**). *Salmonella enterica* synthesizes 5,6-dimethylbenzimidazolyl-(DMB)-alpha-riboside. Why some Firmicutes do not require the canonical DMB activation system to synthesize adenosylcobalamin. **Mol Microbiol** 103(2): 269-281. doi: 10.1111/mmi.13555

24. Claas KR, Parrish JR, Maggio-Hall LA, Escalante-Semerena JC (**2010**). Functional analysis of the nicotinate mononucleotide:5,6-dimethylbenzimidazole phosphoribosyltransferase (CobT) enzyme, involved in the late steps of coenzyme B12 biosynthesis in *Salmonella enterica*. **J Bacteriol** 192(1): 145-154. doi: 10.1128/JB.01159-09

25. Chan CH, Newmister SA, Talyor K, Claas KR, Rayment I, Escalante-Semerena JC (**2014**). Dissecting cobamide diversity through structural

and functional analyses of the base-activating CobT enzyme of *Salmonella enterica*. **Biochim Biophys Acta** 1840(1): 464-475. doi: 10.1016/j.bbagen.2013.09.038

26. O'Donnell BV, Tew DG, Jones OT, England PJ (**1993**). Studies on the inhibitory mechanism of iodonium compounds with special reference to neutrophil NADPH oxidase. **Biochem J** 290 (Pt 1): 41-49. doi: 10.1042/bj2900041

27. Ozsvari B, Bonuccelli G, Sanchez-Alvarez R, Foster R, Sotgia F, Lisanti MP (**2017**). Targeting flavin-containing enzymes eliminates cancer stem cells (CSCs), by inhibiting mitochondrial respiration: Vitamin B2 (Riboflavin) in cancer therapy. **Aging** 9(12): 2610-2628. doi: 10.18632/aging.101351

28. Fonseca MV, Escalante-Semerena JC (**2000**). Reduction of cob(III)alamin to cob(II)alamin in *Salmonella enterica* Serovar Typhimurium LT2. **J Bacteriol** 182(15): 4304-4309. doi: 10.1128/JB.182.15.4304-4309.2000

29. Bulathsinghala CM, Jana B, Baker KR, Postle K (**2013**). ExbB cytoplasmic loop deletions cause immediate, proton motive force-independent growth arrest. **J Bacteriol** 195(20): 4580-4591. doi: 10.1128/JB.00334-13

30. Zgurskaya HI, Nikaido H (**1999**). Bypassing the periplasm: reconstitution of the AcrAB multidrug efflux pump of *Escherichia coli*. **Proc Natl Acad Sci U S A** 96(13): 7190-7195. doi: 10.1073/pnas.96.13.7190

31. Sharma CM, Papenfort K, Pernitzsch SR, Mollenkopf H-J, Hinton JCD, Vogel J (**2011**). Pervasive post-transcriptional control of genes involved in amino acid metabolism by the Hfq-dependent GcvB small RNA. **Mol Microbiol** 81(5): 1144-1165. doi: 10.1111/j.1365-2958.2011.07751.x

32. Hook C, Eremina N, Zaytsev P, Varlamova D, Stoynova N (**2022**). The *Escherichia coli* amino acid uptake protein CycA: regulation of Its synthesis and practical application in l-Isoleucine production. **Microorganisms** 10(3): 1-21. doi: 10.3390/microorganisms10030647

33. Baisa G, Stabo NJ, Welch RA (**2013**). Characterization of *Escherichia coli* D-cycloserine transport and resistant mutants. **J Bacteriol** 195(7): 1389-1399. doi: 10.1128/jb.01598-12

34. Guzman LM, Belin D, Carson MJ, Beckwith J (**1995**). Tight regulation, modulation, and high-level expression by vectors containing the arabinose PBAD promoter. **J Bacteriol** 177(14): 4121-4130. doi : 0.1128/jb.177.14.4121-4130.1995

35. Baker KE, Ditullio KP, Neuhard J, Kelln RA (**1996**). Utilization of orotate as a pyrimidine source by *Salmonella* typhimurium and *Escherichia coli* requires the dicarboxylate transport protein encoded by *dctA*. **J Bacterol** 178(24): 7099-1105. doi: 10.1128/jb.178.24.7099-7105.1996

36. Varadi M, Anyango S, Deshpande M, Nair S, Natassia C, Yordanova G, Yuan D, Stroe O, Wood G, Laydon A, Žídek A, Green T, Tunyasuvunakool K, Petersen S, Jumper J, Clancy E, Green R, Vora A, Lutfi M, Figurnov M, Cowie A, Hobbs N, Kohli P, Kleywegt G, Birney E, Hassabis D, Velankar S (**2022**). AlphaFold protein structure database: massively expanding the structural coverage of protein-sequence space with high-accuracy models. **Nucleic Acids Res** 50(D1): D439-d444. doi: 10.1093/nar/gkab1061

37. Jumper J, Evans R, Pritzel A, Green T, Figurnov M, Ronneberger O, Tunyasuvunakool K, Bates R, Zidek A, Potapenko A, Bridgland A, Meyer C, Kohl SAA, Ballard AJ, Cowie A, Romera-Paredes B, Nikolov S, Jain R, Adler J, Back T, Petersen S, Reiman D, Clancy E, Zielinski M, Steinegger M, Pacholska M, Berghammer T, Bodenstein S, Silver D, Vinyals O, et al. (**2021**). Highly accurate protein structure prediction with AlphaFold. **Nature** 596(7873): 583-589. doi: 10.1038/s41586-021-03819-2

38. Vogl C, Grill S, Schilling O, Stulke J, Mack M, Stolz J (**2007**). Characterization of riboflavin (vitamin B2) transport proteins from *Bacillus subtilis* and *Corynebacterium glutamicum*. **J Bacteriol** 189(20): 7367-7375. doi: 10.1128/JB.00590-07

39. Lee ER, Blount KF, Breaker RR (**2009**). Roseoflavin is a natural antibacterial compound that binds to FMN riboswitches and regulates gene expression. **RNA Biol** 6(2): 187-194. doi: 10.4161/rna.6.2.7727

40. Lewis JA, Escalante-Semerena JC (**2006**). The FAD-dependent tricarballylate dehydrogenase (TcuA) enzyme of *Salmonella enterica* converts tricarballylate into *cis*-aconitate. **J Bacteriol** 188(15): 5479-5486. doi: 10.1128/JB.00514-06

41. Jones H, Venables WA (**1983**). Solubilisation of D-amino acid dehydrogenase of *Escherichia coli* K12 and its re-binding to envelope preparations. **Biochimie** 65(3): 177-183. doi: 10.1016/s0300-9084(83)80082-0

42. Karavaeva V, Sousa FL (**2023**). Modular structure of complex II: An evolutionary perspective. **Biochim Biophys Acta Bioenerg** 1864(1): 148916. doi: 10.1016/j.bbabio.2022.148916

43. Rosenberg G, Yehezkel D, Hoffman D, Mattioli CC, Fremder M, Ben-Arosh H, Vainman L, Nissani N, Hen-Avivi S, Brenner S, Itkin M, Malitsky S, Ohana E, Ben-Moshe NB, Avraham R (**2021**). Host succinate is an activation signal for *Salmonella* virulence during intracellular infection. **Science** 371(6527): 400-405. doi: 10.1126/science.aba8026

44. Powers MJ, Trent MS (**2018**). Phospholipid retention in the absence of asymmetry strengthens the outer membrane permeability barrier to last-resort antibiotics. **Proc Natl Acad Sci U S A** 115(36): E8518-E8527. doi: 10.1073/pnas.1806714115

45. Mewies M, McIntire WS, Scrutton NS (**1998**). Covalent attachment of flavin adenine dinucleotide (FAD) and flavin mononucleotide (FMN) to enzymes: the current state of affairs. **Protein Sci** 7(1): 7-20. doi: 10.1002/pro.5560070102

46. Maher MJ, Herath AS, Udagedara SR, Dougan DA, Truscott KN (**2018**). Crystal structure of bacterial succinate:quinone oxidoreductase flavoprotein SdhA in complex with its assembly factor SdhE. **Proc Natl Acad Sci U S A** 115(12): 2982-2987. doi: 10.1073/pnas.1800195115

47. Walker WH, Singer TP (**1970**). Identification of the covalently bound flavin of succinate dehydrogenase as 8-alpha-(histidyl) flavin adenine dinucleotide. **J Biol Chem** 245(16): 4224-4225. doi: 10.1016/s0021-9258(18)62907-2

48. Copeland RA (**2013**). Reversible Modes of Inhibitor Interactions with Enzymes. Evaluation of Enzyme Inhibitors in Drug Discovery. John Wiley & Sons; pp 57-121.

49. Blat Y (**2010**). Non-competitive inhibition by active site binders. **Chem Biol Drug Des** 75(6): 535-540. doi: 10.1111/j.1747-0285.2010.00972.x

50. Rebholz KL, Northrop DB (**1994**). Kinetics of enzymes with iso-mechanisms: dead-end inhibition of fumarase and carbonic anhydrase II. **Arch Biochem Biophys** 312(1): 227-233. doi: 10.1006/abbi.1994.1303

51. Lewis JA, Horswill AR, Schwem BE, Escalante-Semerena JC (**2004**). The Tricarballylate utilization (*tcuRABC*) genes of *Salmonella enterica* serovar Typhimurium LT2. **J Bacteriol** 186(6): 1629-1637. doi: 10.1128/JB.186.6.1629-1637.2004

52. Lo TC (**1977**). The molecular mechanism of dicarboxylic acid transport in *Escherichia coli* K 12. **J Supramol Struct** 7(3-4): 463-480. doi: 10.1002/jss.400070316

53. Rowland GC, Booth IR (**1984**). D-Alanine transport in *Escherichia coli*. **Biochem Soc Trans** 12(3): 505-505. doi: 10.1042/bst0120505

54. Berkowitz D, Hushon JM, Whitfield HJ, Jr., Roth J, Ames BN (**1968**). Procedure for identifying nonsense mutations. **J Bacteriol** 96(1): 215-220. doi: 10.1128/jb.96.1.215-220.1968

55. Atlas R (**1995**). Handbook of media for environmental microbiology. CRC Press, Boca Raton pp 258.

56. Green MR, Sambrook J (**2012**). Molecular Cloning: A laboratory manual. Cold Spring Harbor Laboratory, Cold Spring Harbor, N.Y.

57. Galloway NR, Toutkoushian H, Nune M, Bose N, Momany C (**2013**). Rapid cloning for protein crystallography using Type IIS restriction enzymes. **Crystal Growth & Design** 13(7):

2833-2839. doi: 10.1021/Cg400171z

58. VanDrisse CM, Escalante-Semerena JC (**2016**). New high-cloning-efficiency vectors for complementation studies and recombinant protein overproduction in *Escherichia coli* and *Salmonella enterica*. **Plasmid** 86: 1-6. doi: 10.1016/j.plasmid.2016.05.001

59. Datsenko KA, Wanner BL (**2000**). One-step inactivation of chromosomal genes in *Escherichia coli* K-12 using PCR products. **Proc Natl Acad Sci U S A** 97(12): 6640-6645. doi: 10.1073/pnas.120163297

60. VanDrisse CM, Parks AR, Escalante-Semerena JC (**2017**). A toxin involved in *Salmonella* persistence regulates its activity by acetylating its cognate antitoxin, a modification reversed by CobB sirtuin deacetylase. **MBio** 8(3): e00708-00717. doi: 10.1128/mBio.00708-17

61. Rocco CJ, Dennison KL, Klenchin VA, Rayment I, Escalante-Semerena JC (**2008**). Construction and use of new cloning vectors for the rapid isolation of recombinant proteins from *Escherichia coli*. **Plasmid** 59(3): 231-237. doi: 10.1016/j.plasmid.2008.01.001

62. Lee JK, Zhao H (**2007**). Identification and characterization of the flavin:NADH reductase (PrnF) involved in a novel two-component arylamine oxygenase. **J Bacteriol** 189(23): 8556-8863. doi: 10.1128/jb.01050-07

63. Markwell JP, Lascelles J (**1978**). Membrane-bound, pyridine nucleotide-independent L-lactate dehydrogenase of *Rhodopseudomonas sphaeroides.* **J Bacteriol** 133(2): 593-600. doi: 10.1128/jb.133.2.593-600.1978

64. Deutch CE (**2004**). Oxidation of 3,4-dehydro-D-proline and other D-amino acid analogues by D-alanine dehydrogenase from *Escherichia coli*. **FEMS Microbiol Lett** 238(2): 383-389. doi: 10.1016/j.femsle.2004.08.001

65. Stead MB, Agrawal A, Bowden KE, Nasir R, Mohanty BK, Meagher RB, Kushner SR (**2012**). RNAsnap: a rapid, quantitative and inexpensive, method for isolating total RNA from bacteria. **Nucleic Acids Res** 40(20): e156. doi: 10.1093/nar/gks680

66. Rocha DJ, Santos CS, Pacheco LG (**2015**). Bacterial reference genes for gene expression studies by RT-qPCR: survey and analysis. **Antonie Van Leeuwenhoek** 108(3): 685-693. doi: 10.1007/s10482-015-0524-1

67. Livak KJ, Schmittgen TD (**2001**). Analysis of relative gene expression data using real-time quantitative PCR and the 2(-Delta Delta C(T)) Method. **Methods** 25(4): 402-408. doi: 10.1006/meth.2001.1262
